# Supplementary material for: Supporting people with type 2 diabetes in effective use of their medicine through mobile health technology integrated with clinical care (SuMMiT-D Feasibility): a randomised feasibility trial protocol
Source: BMJ Open. 2019 Dec 29;9(12):e033504. doi: 10.1136/bmjopen-2019-033504 (PMC6937131; doi:10.1136/bmjopen-2019-033504)
Supplement: Supplementary data [file bmjopen-2019-033504supp001.pdf]

Date and version No: v0.65 6 DEC 2019

# Supplementary Appendix

This appendix has been provided by the authors to give readers additional information about their work.

Supplement to: Supporting people with type 2 diabetes in effective use of their medicine through a system comprising mobile health technology integrated with clinical care compared with usual care (SuMMiT-D Feasibility): a randomised feasibility trial protocol



Farmer A, *et al. BMJ Open* 2019; 12:e033504. doi: 10.1136/bmjopen-2019-033504

Date and version No: v0.65 6 DEC 2019

# Appendix 1.

## Examples of SMS text messages used in SuMMiT-D feasibility

| Target and category of message | BCT/ Belief or concern | Example messages |
|---|---|---|
| Medication adherence, BCT | 1.4 Action Planning | Plan when, where and how you are going to take your medication. |
| Medication adherence, BCT | 15.1 Verbal persuasion about capability | If you are struggling with your diabetes tablets then don't worry, you will be able to master it in time. You will get on top of it. |
| Medication adherence, BCT | 7.1 Prompts/ cues | It can be difficult to remember to take your tablets. Why not set an alarm to remind you to take them? |
| Medication adherence, BCT | 2.3 Self-monitoring | Find a way to split your tablets into days so you notice when you have forgotten to take your tablets |
| Medication adherence, BCT | 3.2 Social support (practical) | How often do you forget to take your tablets? Take control. Ask your friends and family members to help. Their reminders could help you to improve your diabetes |
| Medication adherence, BCT | 15.2 Mental rehearsal of successful performance | Visualise in detail how you will take your tablets tomorrow. This will make it easier when you actually take them |
| Medication adherence, BCT | 3.3 Social support (emotional) | If you're not taking your tablets as often as you should, try discussing your feelings with someone. |
| Medication adherence, BCT | 15.2 Mental rehearsal of successful performance | Think about situations where taking tablets was easy. How could you make your everyday tablet taking like this? |
| Medication adherence, beliefs and concerns | G Healthcare system related concerns | Lots of questions? Check who the best person to see might be |
| Diet management | Signposting | Stuck for new ideas? You can search recipes for mains, desserts and snacks online at Diabetes.org.uk |

Glossary: BCT- Behaviour Change Technique



Farmer A, *et al. BMJ Open* 2019; 12:e033504. doi: 10.1136/bmjopen-2019-033504

Date and version No: v0.65 6 DEC 2019

## Appendix 2 Beliefs and Ideas questionnaire



Farmer A, *et al. BMJ Open* 2019; 12:e033504. doi: 10.1136/bmjopen-2019-033504

**Participant ID:** __ __ / __ __ __ / __ __ __ __

**Date Completed:** DD / MMM / YYYY

# SuMMiT-D Feasibility

## Ideas and concerns about your diabetes treatment – Baseline

***(Questionnaire to be adapted for electronic use as well)***

This survey is about what you think and feel about your diabetes tablets, as well as how you approach taking them. There are no right or wrong answers. We know some of the items might be difficult to answer, but please choose the answer that best describes what you think. When you are completing the survey please think about the tablets you take for your diabetes.

| | | Strongly disagree | Disagree | Neither agree nor disagree | Agree | Strongly Agree |
|---|---|---|---|---|---|---|
| 1. | It is easy to take my diabetes tablets as prescribed | ☐ | ☐ | ☐ | ☐ | ☐ |
| 2. | I am confident that I can take my diabetes tablets as prescribed | ☐ | ☐ | ☐ | ☐ | ☐ |
| 3. | Without my diabetes tablets I would be very ill | ☐ | ☐ | ☐ | ☐ | ☐ |
| 4. | My health in the future will depend on my diabetes tablets | ☐ | ☐ | ☐ | ☐ | ☐ |
| 5. | I sometimes worry about the long-term effects of my diabetes tablets | ☐ | ☐ | ☐ | ☐ | ☐ |
| 6. | My diabetes tablets are a mystery to me | ☐ | ☐ | ☐ | ☐ | ☐ |
| 7. | I want to take my diabetes tablets as prescribed every day over the next 3 months | ☐ | ☐ | ☐ | ☐ | ☐ |
| 8. | I will take my diabetes tablets as prescribed every day over the next 3 months | ☐ | ☐ | ☐ | ☐ | ☐ |



Farmer A, *et al. BMJ Open* 2019; 12:e033504. doi: 10.1136/bmjopen-2019-033504

**Participant ID:** ___ ___ / ___ ___ ___ / ___ ___ ___ ___

| | | Strongly disagree | Disagree | Neither agree nor disagree | Agree | Strongly Agree |
|---|---|---|---|---|---|---|
| 9. | Taking my diabetes tablets as prescribed is something I do without thinking. | ☐ | ☐ | ☐ | ☐ | ☐ |
| 10. | Taking my diabetes tablets as prescribed is something I do without noticing that I do | ☐ | ☐ | ☐ | ☐ | ☐ |
| 11. | I am confident that I am able to take my diabetes tablets as prescribed even when something disrupts my routine | ☐ | ☐ | ☐ | ☐ | ☐ |
| 12. | I am confident that I can take my diabetes tablets as prescribed even when I feel well | ☐ | ☐ | ☐ | ☐ | ☐ |
| 13. | If I don't take my diabetes tablets for any reason, I am confident that I am able to start taking them again even if I feel no different to when I was not taking them | ☐ | ☐ | ☐ | ☐ | ☐ |
| 14. | If I don't take my diabetes tablets for any reason, I can start taking them again even if I had not taken them for a couple of days | ☐ | ☐ | ☐ | ☐ | ☐ |
| 15. | I have made a detailed plan for when to take my diabetes tablets | ☐ | ☐ | ☐ | ☐ | ☐ |
| 16. | I have made a detailed plan about exactly where to take my diabetes tablets | ☐ | ☐ | ☐ | ☐ | ☐ |
| 17. | I have made a detailed plan for what to do to help me take my diabetes tablets as prescribed when facing barriers to do so | ☐ | ☐ | ☐ | ☐ | ☐ |

**Participant ID:** ___ ___ / ___ ___ ___ / ___ ___ ___ ___

| | | Strongly disagree | Disagree | Neither agree nor disagree | Agree | Strongly Agree |
|---|---|---|---|---|---|---|
| 18. | I have made a detailed plan for how to deal with unpleasant side effects of taking my diabetes tablets as prescribed | ☐ | ☐ | ☐ | ☐ | ☐ |
| 19. | During the last 4 weeks I consistently monitored when, where, and how I took my diabetes tablets | ☐ | ☐ | ☐ | ☐ | ☐ |
| 20. | During the last 4 weeks I really tried hard to take my diabetes tablets as prescribed | ☐ | ☐ | ☐ | ☐ | ☐ |
| 21. | I use things around me to help me to take my diabetes tablets as prescribed (e.g. notes, phone reminders) | ☐ | ☐ | ☐ | ☐ | ☐ |
| 22. | I change things around me to prompt me to take my diabetes tablets as prescribed | ☐ | ☐ | ☐ | ☐ | ☐ |
| 23. | I have had somebody help me to take my diabetes tablets as prescribed | ☐ | ☐ | ☐ | ☐ | ☐ |
| 24. | I have felt supported in taking my diabetes tablets as prescribed | ☐ | ☐ | ☐ | ☐ | ☐ |
| 25. | Given the effort I have put into taking my diabetes tablets, I am content with my diabetes control | ☐ | ☐ | ☐ | ☐ | ☐ |
| 26. | I am content with what I have experienced as a result of taking my diabetes tablets | ☐ | ☐ | ☐ | ☐ | ☐ |
| 27. | It is likely that I will develop complications, or experience | ☐ | ☐ | ☐ | ☐ | ☐ |

**Participant ID:** ___ ___ / ___ ___ ___ / ___ ___ ___ ___

| | | Strongly disagree | Disagree | Neither agree nor disagree | Agree | Strongly Agree |
|---|---|---|---|---|---|---|
| | worse complications from my diabetes if do not take my tablets | | | | | |
| 28. | I feel very at risk of developing complications, or experiencing worsening of existing complications from my diabetes if I do not take my tablets | ☐ | ☐ | ☐ | ☐ | ☐ |

If you have any comments about this survey, please leave them below.

| |
|---|
| |

**Thank you for completing this survey.**

**Please return to the SuMMiT-D Feasibility study team in the envelope provided.**

**If you have any questions, please contact: xxxxxxxxxxx**



Farmer A, *et al. BMJ Open* 2019; 12:e033504. doi: 10.1136/bmjopen-2019-033504

Date and version No: v0.65 6 DEC 2019

## Appendix 3

**Members of the SuMMiT-D Feasibility Trial Collaborative Group:**

### Writing Group

A. J. Farmer[1], J.Allen[1], Y.K. Bartlett[2], P. Bower[2], Y. Chi[1], D.P. French[2], B Gudgin[3], E. Holmes[4], R. Horne[5], D.A. Hughes[4], C. Kenning[2], L. Locock[6], J. McSharry[7], L. Miles[2], N. Newhouse[1], R. Rea[1], E. Riga[1], L. Tarassenko[1], C. Velardo[1], N. Williams[1], V. Williams[1], L-M Yu[1].

Author affiliations:
1. University of Oxford, Oxford UK.
2. University of Manchester, Manchester UK.
3. Patient Representative, Oxfordshire, UK.
4. Bangor University, Bangor UK.
5. University College London, UK.
6. University of Aberdeen, Aberdeen UK
7. National University of Ireland, Galway, Eire.

### Programme Steering Committee

***Independent Members***

Chair: K. Khunti; C. Weir, F. Sniehotta, S. England

***Investigators***

A. Farmer; D.P. French, L-M. Yu

***Observers***

T. Hutchinson (Funder), E. Chick (Sponsor), C. Velardo, E. Riga, S. M. Robinson

### Trial Management Team

J. Allen, Senior Trial Manager; E. Riga, Trial Manager; S. M. Robinson, Trial Coordinator; C. Kenning, Regional Coordinator

### Statistics

L-M. Yu, Lead Trial Statistician; N. Williams, Senior Statistician; M. Shanyinde, Statistician

### Data Management

S. Kelly, Senior Data Manager; L. Engonidou, Data Manager

### IT, Programming and Engineering

D. Judge, Senior IT and Programming Manager; C. Velardo, Senior Research Fellow; Y. Chi, Post-doctoral Research Assistant.

**Research Nurses**

H. Rutter, Senior Research Nurse; S. Squires, Research Nurse; B. Mundy, Research Nurse; K. Madronal, Research Nurse; I. Noel, Research Nurse; B. I'Anson, Research Nurse; P. Whitbread, Research Nurse

**PPI Members**

Chair: B. Gudgin; G. Moultrie, M. Simpson, P. Baller, R. Dawett, D. Akker, M. Mistry, S. Mulholland, J. Nugent, G. Sides, D. Parkinson, S. McPhillips

**Acknowledgements**
In addition to the team members acknowledged above, we would also like to acknowledge the help of the following clinical research networks (CRNs) and participating GP practices supporting recruitment:

| **Practice Name** |
|---|
| NIHR CRN: Thames Valley & South Midlands |
| South Oxford Health Centre |
| White Horse Medical Practice |
| Botley Medical Centre |
| The Rycote Practice |
| Milman Road Health Centre/Milman Medical |
| Broadshires Health Centre |
| Bicester Health Centre |
| NIHR CRN: West Midlands |
| Eve Hill Medical Practice |
| Spring Gardens Group Medical Practice |
| Burbury Medical Centre |
| NIHR CRN: South West Peninsula |
| Brunel Medical Practice |
| Newquay Health Centre |
| Glastonbury Surgery |
| NIHR CRN: Greater Manchester |
| Block Lane Surgery |
| Ashcroft Surgery |
| West Timperley Medical Centre |
| Chorlton Family Practice |
| Washway Road Medical Centre |
| Market Street Medical Practice |

Trial Sponsor: University of Oxford. Contact Clinical Trials and Research Governance, Churchill Hospital, Oxford, UK.
