# Supplementary material for: Supporting people with type 2 diabetes in effective use of their medicine through mobile health technology integrated with clinical care (SuMMiT-D Feasibility): a randomised feasibility trial protocol
Source: BMJ Open. 2019 Dec 29;9(12):e033504. doi: 10.1136/bmjopen-2019-033504 (PMC6937131; doi:10.1136/bmjopen-2019-033504)
Supplement: Author's manuscript [file bmjopen-2019-033504.draft_revisions.pdf]

BMJ Open is committed to open peer review. As part of this commitment we make the peer review history of every article we publish publicly available.

When an article is published we post the peer reviewers' comments and the authors' responses online. We also post the versions of the paper that were used during peer review. These are the versions that the peer review comments apply to.

The versions of the paper that follow are the versions that were submitted during the peer review process. They are not the versions of record or the final published versions. They should not be cited or distributed as the published version of this manuscript.

BMJ Open is an open access journal and the full, final, typeset and author-corrected version of record of the manuscript is available on our site with no access controls, subscription charges or pay-per-view fees (http://bmjopen.bmj.com).

If you have any questions on BMJ Open's open peer review process please email info.bmjopen@bmj.com

BMJ Open

Supporting people with type 2 diabetes in effective use of their medicine through mobile health technology integrated with clinical care (SuMMiT-D Feasibility): a randomised feasibility trial protocol

Journal:	*BMJ Open*
Manuscript ID	bmjopen-2019-033504
Article Type:	Protocol
Date Submitted by the Author:	09-Aug-2019

Complete List of Authors:	Farmer, Andrew; University of Oxford, Nuffield Department of Primary Care Health Sciences<br>Allen, Julie; University of Oxford, Nuffield Department of Primary Care Health Sciences<br>Bartlett, Kiera; The University of Manchester, The Division of Psychology and Mental Health<br>Bower, Peter; The University of Manchester, Division of Population Health, Health Services Research & Primary Care<br>Chi, Yuan; University of Oxford, The Institute of Biomedical Engineering<br>French, David; The University of Manchester, The Division of Psychology and Mental Health<br>Gudgin, Bernard; Patient Representative<br>Holmes, EA; Bangor University<br>Horne, Robert; University College London, UCL School of Pharmacy<br>Hughes, Dyfrig; Bangor University, Centre for Health Economics and Medicines Evaluation<br>Kenning, Cassandra; The University of Manchester, Institute of Population Health<br>Locock, Louise; University of Aberdeen, Health Service Research<br>McSharry, Jenny; National University of Ireland Galway, Health Behaviour Change Research Group, School of Psychology<br>Miles, Lisa; The University of Manchester, Division of Psychology and Mental Health<br>Newhouse, Nikki; University of Oxford, Nuffield Department of Primary Care Health Sciences<br>Rea, Rustam ; University of Oxford, Nuffield Department of Primary Care Health Sciences<br>Riga, Evgenia; University of Oxford, Nuffield Department of Primary Care Health Sciences<br>Tarassenko, Lionel; University of Oxford, Institute of Biomedical Engineering<br>Velardo, Carmelo; University of Oxford, Institute of Biomedical Engineering<br>Williams, Nicola; University of Oxford, Nuffield Department of Primary Care Health Sciences<br>Williams, Veronika; University of Oxford, Nuffield Department of Primary Care Health Sciences<br>Yu, Ly-Mee; University of Oxford, Nuffield Department of Primary Care

	Health Sciences
Keywords:	type 2 diabetes, digital health, behaviour change intervention, PRIMARY CARE, randomised controlled trial, process evaluation

SCHOLARONE™
Manuscripts

For submission to BMJ Open

Supporting people with type 2 diabetes in effective use of their medicine through mobile health technology integrated with clinical care (SuMMiT-D Feasibility): a randomised feasibility trial protocol

The SuMMiT-D Collaborative Group

Andrew J. Farmer[1], Julie Allen[1], Y.Kiera Bartlett[2], Peter Bower[2], Yuan Chi[1], David P. French[2], Bernard Gudgin[3], Emily Holmes[4], Rob Horne[5], Dyfrig A. Hughes[4], Cassandra Kenning[2], Louise Locock[6], Jennifer McSharry[7], Lisa Miles[2], Nikki Newhouse[1], Rustam Rea[1], Jenny Riga[1], Lionel Tarassenko[1], Carmelo Velardo[1], Nicola Williams[1], Veronika Williams[1], Ly-Mee Yu[1].

Author affiliations:
1. University of Oxford, Oxford UK.
2. University of Manchester, Manchester UK.
3. Patient Representative, Oxfordshire, UK.
4. Bangor University, Bangor UK.
5. University College London, UK.
6. University of Aberdeen, Aberdeen UK
7. National University of Ireland, Galway, Eire.

Abstract: 300
Main Text: 3925/4,000

Figures: 1

Date and version No: 09-AUG-2019; v0.58

Correspondence to: Andrew Farmer, Nuffield Department of Primary Care Health Sciences, Woodstock Road, Oxford OX2 6GG. andrew.farmer@phc.ox.ac.uk
Tel: +44 1865 289280

Key Words
Type 2 diabetes, digital health, behavioural change intervention, primary care, feasibility study, process evaluation, randomized controlled trial.

For peer review only

Abstract

Introduction Type 2 diabetes is common, affecting over 400 million people worldwide. Risk of serious complications can be reduced through use of effective treatments and active self-management. However, people are often concerned about starting new medicines and face difficulties in taking them regularly. Use of brief messages to provide education and support self-management, delivered through mobile phone-based text-messages can be an effective tool for some long-term conditions. We have developed messages aiming to support patients' self-management of type 2 diabetes in the use of medications and other aspects of self-management, underpinned by theory and evidence. The aim of this trial is to determine the feasibility of a large-scale clinical trial to test the effectiveness and cost-effectiveness of the intervention, compared to usual care.

Methods and analysis The feasibility trial will be a multi-centre individually randomised, controlled trial in primary care recruiting adults (≥35 years) with type 2 diabetes in England. Consenting participants will be randomised to receive short text-messages three times a week with messages designed to produce change in medication adherence or non-health related messages for six months. The aims are to test recruitment methods, retention to the study, the feasibility of data collection and the mobile-phone and web-based processes of a proposed definitive trial and to refine the text messaging intervention. Data, including patient reported measures, will be collected online at baseline and the end of the six-month follow-up period. With 200 participants (100 in each group), this trial is powered to estimate 80% follow up within 95% confidence intervals of 73.8% to 85.3%. The analysis will follow a pre-specified plan.

Ethics and Dissemination Ethics approval was obtained from the West of Scotland Research Ethics Committee 05. The results will be disseminated through conference presentations, peer-reviewed journals and will be published on the trial website: www.summit-d.org

SuMMiT-D (SUpport through Mobile Messaging and digital health Technology for Diabetes)

Trial Registration

ISRCTN13404264; date of registration 10 October 2018

For peer review only

Article Summary

Strengths and limitations of this study

1. The use of brief messages to support self-management and provide motivation and education, delivered through mobile phone-based text-messages can be an effective tool for some long-term conditions.
2. There are a few trials testing the impact of brief messaging in type 2 diabetes, but they do not have systematically developed interventions targeting a range of behaviour change techniques.
3. The objective of the SuMMiT-D Feasibility trial is to test recruitment and randomisation of participants, and collection of proposed primary and secondary outcome data in planning for a large, outcomes based, randomised controlled trial.
4. The messages evaluated in this study are (i) targeted at constructs identified as being related to medication adherence by systematic reviews of the literature, and (ii) demonstrated to have fidelity to intended behaviour change techniques (as rated by experts) and acceptability to target population (as rated by patients)
5. Potential mechanisms for the action of the brief messages will be assessed through changes in constructs that are related to medication-taking behaviour, questions based on the Technology Acceptance model, and self-report medication adherence.

Introduction

Type 2 diabetes is one of the most common long-term conditions affecting 422 million people worldwide[1] and 4.7 million people in the UK.[2] It can lead to major complications including cardiovascular disease, renal failure and neuropathy.[3] The global burden of diabetes is projected to reach up to 2.2% of global gross domestic product,[4] and many of these costs are due to preventable complications. Prevention includes the use of treatments of proven efficacy[5,6] alongside supporting self-management.[7] However, concerns about medicines and difficulties in taking them regularly, whether intentional or unintentional, are common[8] and lead to poor diabetes control and increased use of health resources.[9] The cost of non-adherence to diabetes medication in the United Kingdom has been estimated at £100 million per year in avoidable treatment costs.[10] A wide range of services are available to support people in better use of their medicines, but evidence of their effectiveness and cost-effectiveness is weak. These services are often targeted at particular groups of individuals and are frequently designed as "one-off" interventions. Understanding and improving this situation could make a major contribution to health and health-care costs.

Systematic reviews do not provide strong evidence to support the wider use of current approaches to developing interventions to improve adherence.[11] Brief messages delivered at a wide scale and low cost via digital health systems added to usual care have been shown to be effective in improving health for some conditions and are a promising approach to the problem.[12]

Systematic reviews of text messages to support patients to adhere to treatment, and of mobile health interventions in diabetes, identify some effective interventions. There are a few trials testing the impact of brief messaging in type 2 diabetes, but they do not have systematically-developed interventions based on theory and evidence and are at risk of bias.[12,13] Recent trials of text-messaging for cardiovascular risk prevention and blood pressure lowering have shown clinically relevant changes in outcomes compared with usual care.[14,15]

In addition, there is substantial evidence that tailored interventions are more effective than generic interventions.[16] Tailored interventions may be seen by recipients as more personally relevant, so they will be more likely to attend to, read, understand, and act on them. In addition, tailored interventions are designed to change determinants of the target behaviour that are relevant to particular individuals

For peer review only

or to small subgroups of individuals; they therefore more precisely target the determinants of the individual's behaviour.

SuMMiT-D (SUpport through Mobile Messaging and digital health Technology for Diabetes) is a programme of work comprised of three phases: formative work, a feasibility trial and a large scale, effectiveness randomised controlled trial of a mobile phone-based system intended to deliver brief, tailored, behaviour-change messages to people with type 2 diabetes focusing on use of medication. In the formative work for this trial, we identified theoretical constructs and features of intervention content found to be associated with medication adherence in patients with type 2 diabetes and mapped these onto a standard taxonomy for behaviour change techniques (BCTs), that is, active ingredients of interventions used to promote behavioural change[17,18]. We then developed a large set of messages to target each BCT, and examined which types of messages are most useful and easy to understand for people starting and taking an oral diabetes medicine, and the extent to which it might be helpful for patients to decide on the types of messages they want to receive. We received input from approximately 300 patients with type 2 diabetes and healthcare professionals caring for this patient population. Preliminary findings of this trial development work were used to develop this feasibility trial with scope for further refinement of the system for the main clinical trial.

The primary objective of the SuMMiT-D Feasibility trial is to test recruitment and randomisation of participants to the trial. We will test collection of planned primary and secondary outcome data in planning for a large, outcomes-based, randomised controlled trial. We will assess the feasibility and acceptability of the intervention for patients and healthcare professionals; the willingness of participants to be randomised; follow-up rates; resource use and trial procedures. We will also carry out a process evaluation of how the system is used and refine the way it is embedded within usual care. Potential mechanisms for the action of the brief messages will be assessed through changes in hypothesised health psychology constructs relating to use of medication, self-report medication adherence,[19] and questions based on the Technology Acceptance model.[20]

Methods and analysis

The SuMMiT-D Feasibility trial protocol is reported according to SPIRIT and EMERGE recommendations. [21,22]

For peer review only - http://bmjopen.bmj.com/site/about/guidelines.xhtml

Patient and Public Involvement

Patient members of the public are integral to this trial. A panel of eleven patient and public involvement (PPI) members with type 2 diabetes was set up and continues to inform our work, reviewing all patient documentation and initial research findings from the formative work, and to support the development of the intervention.

All patient facing documents for the SuMMiT-D feasibility trial, including, the participant information sheet, informed consent form, posters, user guides, and website were reviewed by PPI panel members. The panel has been kept up to date with frequent trial updates in the form of an email newsletter, also published on the trial website (www.summit-d.org). The results of the study will be made available to trial participants, PPI panel members and participating GP practices on the trial website.

Trial Design

SuMMiT-D Feasibility is a primary care based, two-arm, individually randomised controlled, parallel group trial aiming to recruit a total of 200 patients across twenty general practice sites in England. Patients with type 2 diabetes will be randomly allocated to receive an individually tailored Short Messaging System (SMS) text-messaging based intervention for 26 weeks that aims to encourage and support them in developing a habit of taking their medication as intended (that is, to promote effective implementation of dosing and treatment continuation)[23] and provides hints and tips to help them with other aspects of living with the condition alongside usual care (treatment arm), or to usual care with the addition of infrequent non-health related messages (control arm) (Figure 1).

Intervention

Intervention arm: Condition-specific tailored text messaging system and usual care

Participants assigned to usual care will receive brief health related SMS text messages, based on systematic review of the evidence identifying determinants of medication taking behaviour.[18] Messages were developed based on systematic review evidence by experts,[18] and were refined in an iterative process of ensuring acceptability based on patient feedback and demonstration of fidelity to intended behaviour change determinants, as rated by an independent group of experts.[24]

For peer review only - http://bmjopen.bmj.com/site/about/guidelines.xhtml

The intervention is a digital health system with the following components:

i. Participants will be sent up to four automated text-messages per week with an average frequency of three per week relating to diabetes management and use of medicine.

ii. The library of text-messages uses different behaviour change techniques to target health-related behaviour change relating to use of medicines, as well as messages targeting other aspects of diabetes care (including diet and exercise).

iii. The types of messages sent will be tailored to participants based on time since starting new glucose, blood pressure and cholesterol-lowering medication and current smoking status.

iv. Frequency of messages received using a particular type of behaviour change technique can be modified based on a participant's response to individual messages received.

v. The style of messages will be patient-centred and will encourage patients to seek further relevant information (including the use of links where possible to selected external websites e.g. Diabetes UK).

Control arm: Usual care and one non-health-related message a month

Participants assigned to usual care will receive non-health related text-messages at a frequency of approximately one every four weeks. Care will otherwise not differ from usual care.

Outcomes

The primary outcome is the rate of recruitment to randomisation of participants to the trial. We aim to recruit 200 people in three months. We will measure recruitment against planned recruitment rates for the proposed main trial and number of people showing an interest in the trial and not proceeding or those who withdraw from the control group and give a reason.

Secondary outcomes focus on feasibility of collection of clinical and economic measurement data for the proposed main trial. They include completeness of HbA_{1c}, systolic blood pressure and total to HDL ratio records, and retention rates, proportion of expected medication use data available, and completion of resource use and health outcome questionnaires. Data on changes in clinical parameters will be reported.

Procedures and assessments

Participants who express interest in taking part in the trial will be screened by the trial team and will consent and submit their baseline questionnaires either online or

For peer review only - http://bmjopen.bmj.com/site/about/guidelines.xhtml

on paper according to their preference. Participants will be randomised by the trial team and will receive messages for 26 weeks from randomisation to final follow-up. All participants will be asked to complete the same set of questionnaires at baseline and at the end of their 26-week follow-up period. Medical notes reviews will be conducted at baseline, six- and 18- months from randomisation.

Recruitment

Potential participants will be identified through general practices in England. Participating practices fall under four Clinical Research Networks (CRNs): Thames Valley & South Midlands, Greater Manchester, West Midlands and South West Peninsula.

Healthcare professionals will screen their type 2 diabetes clinic lists and will invite eligible patients. Patients will receive an invitation letter and summary information leaflet. Eligible patients may also be contacted by phone, email or text by the practice team up to three times.

Expressions of interest

People interested in taking part can send their full name by SMS text-message to the trial team to register their interest. If potential participants have any difficulties in registering their interest in the trial, they will also be able to contact the team via phone or email and will receive support in registering as required.

Screening assessment

Following an expression of interest, further information about the study will be provided by email or by post as requested. Eligibility will be confirmed by phone.

Inclusion criteria

Eligible participants are ≥35 years of age, taking oral glucose lowering treatment, blood pressure lowering treatment or lipid-lowering treatment either alone or in combination. They have access to a mobile phone and are able, if necessary, with help (e.g. relative, friend, neighbour), to send, understand and retrieve brief SMS text-messages in the English language. Participants who are using insulin treatment without also concomitant use of oral glucose lowering treatment; who are pregnant, within three months post-partum or planning pregnancy during the course of the trial; have a serious medical condition that, in the opinion of the investigator, makes them ineligible; have been admitted to hospital within the last three months for hyper- or hypoglycaemia, are ineligible.

For peer review only

Informed consent

Participants will provide consent either on-line or on paper.

Baseline and follow up assessments

The following questionnaires will be administered (online or by post) at the baseline assessment: the Medication Adherence Report Scale (MARS) self-report scale,[19] the EuroQol 5-Dimension, 5-Level (EQ-5D-5L) scale,[25] a set of measures developed for the study based on the technology acceptance model,[20] and a healthcare utilisation record to allow healthcare resource use to be costed. A further set of measures based on a logic model based on the Health Action Process Approach has been developed to show the hypothesised mechanism of action (Supplementary Figure 1).[26] The logic model shows anticipated mechanisms of action and how the constructs measured in the feasibility study (and which are targeted by the BCTs contained within the messages) relate to the main behavioural outcome of habitual medication taking.

All self-report data will be collected either online or on paper according to the participant's preference. The same measures are collected at 26 weeks after randomisation alongside clinical record data.

Randomisation

Participants will be randomised after consent and when all baseline assessments have been completed. Participants will be allocated in a 1:1 ratio to either the intervention or the control arm. Randomisation will be done using a validated secure web-based randomisation programme (Sortition) provided by the University of Oxford Primary Care Clinical Trials Unit (PC-CTU). Allocation will be carried out with a non-deterministic minimisation algorithm to ensure groups are balanced for important baseline prognostic and other factors: study site, age (<65/≥65 years), gender (male/female), duration of diabetes (<5 years/≥5 years), number of medications (<5/≥5). The allocated intervention will be implemented directly by the platform on which the digital health system is run. Apart from the qualitative research team and the engineering team, allocation is blinded to all other trial and healthcare staff. Due to the nature of the study, unblinding is not required during the trial.

Discontinuation of intervention or withdrawal from trial

Participants can withdraw from the trial at any time. Participants can also choose to Pause or Stop receipt of text messages by sending a text-message or contacting the

For peer review only - http://bmjopen.bmj.com/site/about/guidelines.xhtml

trial office by telephone or post. Adverse events are collected and serious unexpected adverse events related to the intervention are determined by the chief investigator and reported in line with local procedures.

Statistical Analysis

Power

With 200 participants (100 in each group) the feasibility trial is powered to estimate 80% follow up within 95% confidence intervals of 73.8% to 85.3%.

Analysis

The primary outcome will be the number of patients recruited to randomisation as a proportion (with 95% confidence interval) of the target recruitment number. Secondary outcomes will be reported overall and separately by allocated arm.

An intention-to-treat approach will be used for an exploratory analysis of secondary outcomes by allocated arm. Continuous outcomes will be analysed using an analysis of covariance adjusting for minimisation factors. Results will be presented as an adjusted difference in means with 95% confidence interval. Binary outcomes will be analysed in a similar way using log binomial regression models (adjusting for minimisation factors). Results will be presented as relative risks with 95% confidence intervals. There will be no formal assessment of treatment efficacy for this feasibility study.

Analysis of health economic measures

Data collected on healthcare utilisation will be assessed for completeness. The items recorded include patient contacts with healthcare professionals, use of primary care services, hospital outpatient appointments, accident and emergency visits, and inpatient stays, prescribed and purchased medications, and personal social services. The proportion of complete and returned questionnaires will be reported. Total costs associated with each item of resource will be estimated based on unit costs from standard sources,[27,28] and presented descriptively.

Responses to the EQ-5D-5L questionnaire will also be examined for completeness and reported by proportion of returned questionnaires and items with a valid response. Health utility values corresponding to patients' EQ-5D-5L profiles will be estimated based on English tariff scores,[29] and presented descriptively.

For peer review only

Process evaluation

We will perform a mixed-methods process analysis, consisting of a qualitative element and a quantitative element. The qualitative process evaluation will examine the experience of participants and health professionals in their use of and implementation of the messaging system. Longitudinal interviews with up to 30 participants will take place at the beginning of the trial and at end of 26-week follow-up. Additionally, 12 to 30 healthcare professionals will be invited to share their experiences of taking part in this trial.

Interviews with trial participants

Participants who consent to taking part in the embedded qualitative study will be purposefully selected by characteristics including age, gender, length of time of diabetes and type of medication, and invited to share their views on the messaging system, and to provide insight into how it was implemented in daily life, identify issues around potential attrition and inform final procedures for the main trial.

Interviews and/or focus groups with healthcare professionals

Healthcare staff from practices taking part in the trial will be invited to take part in focus groups or qualitative interviews to share their experience of recruiting for this trial, and how the intervention was implemented in routine clinical care. Eligible staff will be those with a potential role in the implementation of a text-message system or who have contact with patients with type 2 diabetes. These will include GPs, practice nurses, receptionists and healthcare assistants.

Analysis of qualitative data: trial participants

Thematic analysis will be used inductively to identify key issues, focussing on similarities and differences identified in themes to understand the issues that are important to the participants.[30] We will use NVivo and mind-mapping software to code data, identify themes and interpret these and their relationship. The data analysis will be inductive.

Analysis of qualitative data: healthcare professionals

We will follow a semi-structured topic guide based on the core constructs of extended normalization process theory (NPT):[31] Coherence, Reflexive Monitoring, Cognitive Participation and Collective Action to ensure key areas are covered while allowing flexibility to follow up topics introduced by participants. NVivo software will

For peer review only

be used to facilitate data organisation, analysis and the development of themes. The analysis will use a thematic approach informed by NPT.[30]

Quantitative process analysis

To facilitate understanding of mechanism of the intervention, the effect of the intervention on the set of measures based on the logic model will be analysed using a per-protocol analysis for responders, according to allocated group. This will identify if these measures have been affected by the intervention. A further analysis will examine how these measures in the logic model (controlling for baseline) predict change in self-reported medication adherence using hierarchical multiple regression techniques.[32] This analysis will thereby assess extent of association between measures in logic model and beliefs associated with medication adherence.[33] These analyses will be used in conjunction with the qualitative analyses described above to inform decisions about selection of SMS messages relating to particular BCTs, and potentially refinement of the logic model and selection of measures for the full trial.

Data Management

All trial data will be entered on electronic case report forms. The clinical database is built on REDCap (Research Electronic Data Capture), a secure, web-based application designed to support data capture for research studies.[34]

Ethics

The trial will be conducted according to the principles of the Declaration of Helsinki and in accordance with other relevant national guidelines, regulations, acts and using Good Clinical Practice guidelines. The University of Oxford sponsors the trial.

The protocol, informed consent form, participant information leaflet and all other patient-facing material have been submitted and approved by the West of Scotland Research Ethics Committee 05 (HRA ref: 18/WS/0173). The role of the Trial Steering Committee is taken on by the NIHR Programme Steering Committee.

Results

Recruitment to the SuMMiT-D Feasibility Study began with the first participant randomised on 26 November 2018 and the last participant randomised on 16th April 2019. Reporting of the trial is anticipated in the first quarter of 2020.

For peer review only - http://bmjopen.bmj.com/site/about/guidelines.xhtml

Discussion

SuMMiT-D Feasibility will inform the design of a future large-scale randomised controlled trial, aiming to estimate the clinical and cost effectiveness of the text-messaging intervention and identify difficulties that might be encountered in practice. If effective and implemented in the NHS, this intervention could help reduce the burden of complications and increased costs associated with non-adherence. This research could also offer a model for technology-based self-management support that could be extended to other aspects of diabetes care and other long-term conditions.

Author contributions

A. J. Farmer,	andrew.farmer@phc.ox.ac.uk
J. Allen,	julie.allen@phc.ox.ac.uk
Y.K. Bartlett,	kiera.bartlett@manchester.ac.uk
P. Bower,	peter.bower@manchester.ac.uk
Y. Chi,	yuan.chi@eng.ox.ac.uk
D.P. French,	david.french@manchester.ac.uk
B. Gudgin	bernard_gudgin@compuserve.com
E. Holmes,	e.holmes@bangor.ac.uk
R. Horne	r.horne@ucl.ac.uk
D.A. Hughes	d.a.hughes@bangor.ac.uk
C. Kenning,	cassandra.kenning@manchester.ac.uk
L. Locock,	louise.locock@abdn.ac.uk
J. McSharry,	jenny.mcsharry@nuigalway.ie
L. Miles	lisa.miles-2@manchester.ac.uk
N. Newhouse,	nicola.newhouse@phc.ox.ac.uk
R. Rea	rustam.rea@nhs.net
E. Riga,	jenny.riga@phc.ox.ac.uk
L. Tarassenko,	lionel.tarassenko@eng.ox.ac.uk
C. Velardo,	carmelo.velardo@eng.ox.ac.uk
N. Williams,	nicola.williams@phc.ox.ac.uk

For peer review only

V. Williams, veronika.williams@phc.ox.ac.uk

L-M Yu. ly-mee.yu@phc.ox.ac.uk

Each author has contributed significantly to, and is willing to take public responsibility for, one or more aspects of the study. AF, DF, PB, LL, RR, VW, L-MY, JMcS, CV, LT, DH, RH participated integrally in the study design. All authors contributed to implementation of the study protocol. AF and ER drafted the initial manuscript; all other authors provided critical revisions and approved the final version.

Funding This article presents independent research funded by the National Institute for Health Research (NIHR) under its Programme Grants for Applied Research as part of a wider programme of work (RP-PG-1214-20003). The views expressed are those of the authors and not necessarily those of the NHS, the NIHR or the Department of Health and Social Care. The SuMMiT-D research team acknowledge the support of the NIHR through the Clinical Research Networks (AF NIHR Senior Investigator and AF and RR funding from NIHR Oxford Biomedical Research Consortium)

Competing interests All authors have completed the ICMJE uniform disclosure form at www.icmje.org/coi_disclosure.pdf and declare: all authors had financial support from NIHR Programme Grants for Applied Research RP-PG-1214-20003 for the submitted work; no authors reported financial relationships with any organisations that might have an interest in the submitted work in the previous three years; LT reported personal fees from Sensyne Health, CV reported salary support from Sensyne Health and RH reported grants and personal fees from AstraZeneca, personal fees from GSK, personal fees from TEVA, personal fees from Amgen, personal fees from Astellas, personal fees from Abbvie, personal fees from Novartis, from Allergan, outside the submitted work; and is a director of Spoonful of Sugar Ltd, a University College London Business providing consultancy on treatment engagement and patient support programmes to healthcare policy makers, providers and industry..

.

For peer review only

Data

A manuscript with the results of the primary outcome will be published in a peer-reviewed journal. Additional manuscripts will report secondary outcomes and be submitted for publication in peer-reviewed journals. Data will be available from the main trial following completion of the analysis specified in the trial statistical analysis plan on request from the trial data repository (www.SuMMiT-D.org).

References

1. World Health Organization. Global report on diabetes. Geneva: World Health Organization; 2016. 88 p.

2. UK D. Us, diabetes and a lot of facts and stats. London: Diabetes UK; 2019. 48 p.

3. UK Prospective Diabetes Study Group. Overview of 6 years' therapy of type II diabetes: a progressive disease. Diabetes. 1995;44:1249–58.

4. Bommer C, Sagalova V, Heesemann E, Manne-Goehler J, Atun R, Bärnighausen T, et al. Global Economic Burden of Diabetes in Adults: Projections From 2015 to 2030. Diabetes Care. 2018 Apr 20;41(5):963–70.

5. UK Prospective Diabetes Study UKPDS Group. Efficacy of atenolol and captopril in reducing risk of macrovascular and microvascular complications in type 2 diabetes: UKPDS 39. 1998;317:713–20.

6. Colhoun H, Betteridge D, Durrington P, Hitman G, Neil H, Livingstone S, et al. Primary prevention of cardiovascular disease with atorvastatin in type 2 diabetes in the Collaborative Atorvastatin Diabetes Study (CARDS): multicentre randomised placebo-controlled trial. Lancet. 2004;364(1474-547; 9435):685–96.

7. Norris SL, Lau J, Smith SJ, Schmid CH, Engelgau MM. Self-Management Education for Adults With Type 2 Diabetes: A meta-analysis of the effect on glycemic control. 2002;25(7):1159–71.

8. McSharry J, McGowan L, Farmer AJ, French DP. Perceptions and experiences of taking oral medications for the treatment of Type 2 diabetes mellitus: a systematic review and meta-synthesis of qualitative studies. Diabet Med. 2016 Oct;33(10):1330–8.

9. Asche C, LaFleur J, Conner C. A Review of Diabetes Treatment Adherence and the Association with Clinical and Economic Outcomes. Clin Ther. 2011 Jan;33(1):74–109.

10. Salas M, Hughes D, Zuluaga A, Vardeva K, Lebmeier M. Costs of Medication Nonadherence in Patients with Diabetes Mellitus: A Systematic Review and

For peer review only

Critical Analysis of the Literature. Value Health. Blackwell Publishing Inc; 2009 Sep 1;12(6):915–22.

11. Nieuwlaat R, Wilczynski N, Navarro T, Hobson N, Jeffery R, Keepanasseril A, et al. Interventions for enhancing medication adherence. Nieuwlaat R, editor. Cochrane Database SystRev. Chichester, UK: John Wiley & Sons, Ltd; 2014;11:CD000011.

12. Ricci-Cabello I, Bobrow K, Islam SMS, Chow CK, Maddison R, Whittaker R, et al. Examining Development Processes for Text Messaging Interventions to Prevent Cardiovascular Disease: Systematic Literature Review. JMIR mHealth and uHealth. 2019 Mar 29;7(3):e12191.

13. Farmer AJ, McSharry J, Rowbotham S, McGowan L, Ricci-Cabello I, French DP. Effects of interventions promoting monitoring of medication use and brief messaging on medication adherence for people with Type 2 diabetes: a systematic review of randomized trials. Diabet Med. 2015 Oct 15;33(5):565–79.

14. Chow CK, Redfern J, Hillis GS, Thakkar J, Santo K, Hackett ML, et al. Effect of Lifestyle-Focused Text Messaging on Risk Factor Modification in Patients With Coronary Heart Disease: A Randomized Clinical Trial. JAMA : the journal of the American Medical Association. American Medical Association; 2015 Sep 22;314(12):1255–63.

15. Bobrow K, Farmer AJ, Springer D, Shanyinde M, Yu L-M, Brennan T, et al. Mobile Phone Text Messages to Support Treatment Adherence in Adults With High Blood Pressure (SMS-Text Adherence Support [StAR]): A Single-Blind, Randomized Trial. Circulation. 2016 Feb 9;133(6):592–600.

16. Kassavou A, Sutton S. Automated telecommunication interventions to promote adherence to cardio-metabolic medications: meta-analysis of effectiveness and meta-regression of behaviour change techniques. Health Psychology Review. Taylor & Francis; 2017 Sep 12;12(1):25–42.

17. Michie S, Richardson M, Johnston M, Abraham C, Francis J, Hardeman W, et al. The Behavior Change Technique Taxonomy (v1) of 93 Hierarchically Clustered Techniques: Building an International Consensus for the Reporting of Behavior Change Interventions. Ann Behav Med. 2013 Mar 20;46(1):81–95.

18. Long H, Bartlett YK, Farmer AJ, French DP. Identifying Brief Message Content for Interventions Delivered via Mobile Devices to Improve Medication Adherence in People With Type 2 Diabetes Mellitus: A Rapid Systematic Review. J Med Internet Res. 2019;21(1):e10421–19.

19. Horne R, Weinman J. Patients' beliefs about prescribed medicines and their role in adherence to treatment in chronic physical illness. J Psychosom Res. 1999;47(6):555–67.

20. Venkatesh V, Davis FD. A Theoretical Extension of the Technology Acceptance Model: Four Longitudinal Field Studies. Management Science. 2000 Mar 13;46(2):186–204.

For peer review only

21. Chan A-W, Tetzlaff JM, Altman DG, Laupacis A, Gøtzsche PC, Krleža-Jerić K, et al. SPIRIT 2013 statement: defining standard protocol items for clinical trials. American College of Physicians; 2013. pp. 200–7.

22. De Geest S, Zullig LL, Dunbar-Jacob J, Helmy R, Hughes DA, Wilson IB, et al. ESPACOMP Medication Adherence Reporting Guideline (EMERGE). Ann Intern Med. 2018 Jul 3;169(1):30–5.

23. Vrijens B, De Geest S, Hughes DA, Przemyslaw K, Demonceau J, Ruppar T, et al. A new taxonomy for describing and defining adherence to medications. Br J Clin Pharmac. 2012 Apr 5;73(5):691–705.

24. Bartlett K, Farmer A, Rea R, French DP. Developing acceptable brief messages based on behaviour change techniques (BCTs) to encourage medication adherence in people with Type 2 diabetes.

25. Herdman M, Gudex C, Lloyd A, Janssen M, Kind P, Parkin D, et al. Development and preliminary testing of the new five-level version of EQ-5D (EQ-5D-5L). Qual Life Res. Springer Netherlands; 2011 Dec;20(10):1727–36.

26. Schwarzer R. Modeling health behavior change: How to predict and modify the adoption and maintenance of health behaviors. Applied Psychology: an International Review. 57(1):1–29.

27. Curtis L, Burns A. Unit Costs of Health and Social Care 2018. Canterbury; 2018.

28. Joint Formulary Committee. British National Formulary. 77 ed. London.

29. Devlin NJ, Shah KK, Feng Y, Mulhern B, Ben van Hout. Valuing health-related quality of life: An EQ-5D-5L value set for England. Health Econ. John Wiley & Sons, Ltd; 2018 Jan 1;27(1):7–22.

30. Braun V, Clarke V. Using thematic analysis in psychology. Qualitative Research in Psychology. 2006 Jan;3(2):77–101.

31. May C. Towards a general theory of implementation. Implementation Science. Implementation Science; 2013 Feb 13;8(1):1–1.

32. Tabachnik BG, Fidell LS. Using multivariate statistics. 6 ed. London: Pearson; 1996.

33. Nguyen T-M-U, Caze AL, Cottrell N. What are validated self-report adherence scales really measuring?: a systematic review. Br J Clin Pharmac. 2014 Feb 21;77(3):427–45.

34. Harris PA, Taylor R, Thielke R, Payne J, Gonzalez N, Conde JG. Research electronic data capture (REDCap)—A metadata-driven methodology and workflow process for providing translational research informatics support. Journal of Biomedical Informatics. Elsevier Inc; 2009 Apr 1;42(2):377–81.

For peer review only

Figure 1

Trial Profile

For peer review only

Supplementary Appendix

This appendix has been provided by the authors to give readers additional information about their work.

Supplement to: Supporting people with type 2 diabetes in effective use of their medicine through a system comprising mobile health technology integrated with clinical care compared with usual care (SuMMiT-D Feasibility): a randomised feasibility trial protocol

For peer review only

Appendix 1

Members of the SuMMiT-D Feasibility Trial Collaborative Group:

Writing Group

A. J. Farmer[1], J.Allen[1], Y.K. Bartlett[2], P. Bower[2], Y. Chi[1], D.P. French[2], B Gudgin[3], E. Holmes[4], R. Horne[5], D.A. Hughes[4], C. Kenning[2], L. Locock[6], J. McSharry[7], L. Miles[2], N. Newhouse[1], R. Rea[1], E. Riga[1], L. Tarassenko[1], C. Velardo[1], N. Williams[1], V. Williams[1], L-M Yu[1].

<u>Author affiliations:</u>
1. University of Oxford, Oxford UK.
2. University of Manchester, Manchester UK.
3. Patient Representative, Oxfordshire, UK.
4. Bangor University, Bangor UK.
5. University College London, UK.
6. University of Aberdeen, Aberdeen UK
7. National University of Ireland, Galway, Eire.

Programme Steering Committee

Independent Members

Chair: K. Khunti; C. Weir, F. Sniehotta, S. England

Investigators

A. Farmer; D.P. French, L-M. Yu

Observers

T. Hutchinson (Funder), E. Chick (Sponsor), C. Velardo, E. Riga, S. M. Robinson

Trial Management Team

J. Allen, Senior Trial Manager; E. Riga, Trial Manager; S. M. Robinson, Trial Coordinator; C. Kenning, Regional Coordinator

Statistics

L-M. Yu, Lead Trial Statistician; N. Williams, Senior Statistician; M. Shanyinde, Statistician

Data Management

S. Kelly, Senior Data Manager; L. Engonidou, Data Manager

IT, Programming and Engineering

For peer review only

D. Judge, Senior IT and Programming Manager; C. Velardo, Senior Research Fellow; Y. Chi, Post-doctoral Research Assistant.

Research Nurses

H. Rutter, Senior Research Nurse; S. Squires, Research Nurse; B. Mundy, Research Nurse; K. Madronal, Research Nurse; I. Noel, Research Nurse; B. I'Anson, Research Nurse; P. Whitbread, Research Nurse

PPI Members

Chair: B. Gudgin; G. Moultrie, M. Simpson, P. Baller, R. Dawett, D. Akker, M. Mistry, S. Mulholland, J. Nugent, G. Sides, D. Parkinson, S. McPhillips

Acknowledgements
In addition to the team members acknowledged above, we would also like to acknowledge the help of the following clinical research networks (CRNs) and participating GP practices supporting recruitment:

**Practice Name**
NIHR CRN: Thames Valley & South Midlands
South Oxford Health Centre
White Horse Medical Practice
Botley Medical Centre
The Rycote Practice
Milman Road Health Centre/Milman Medical
Broadshires Health Centre
Bicester Health Centre
NIHR CRN: West Midlands
Eve Hill Medical Practice
Spring Gardens Group Medical Practice
Burbury Medical Centre
NIHR CRN: South West Peninsula
Brunel Medical Practice
Newquay Health Centre
Glastonbury Surgery
NIHR CRN: Greater Manchester
Block Lane Surgery
Ashcroft Surgery
West Timperley Medical Centre
Chorlton Family Practice
Washway Road Medical Centre
Market Street Medical Practice

Trial Sponsor: University of Oxford. Contact Clinical Trials and Research Governance, Churchill Hospital, Oxford, UK.

For peer review only - http://bmjopen.bmj.com/site/about/guidelines.xhtml

Appendix 2.

Supplementary Figure 1

Logic model

Title: Logic model showing anticipated mechanisms of action in the SuMMIT-D Feasibility study, based on the Health Action Process Approach

(a) *Footnote*: (a) This construct includes both beliefs about the necessity of taking medication, and concerns about taking that medication.

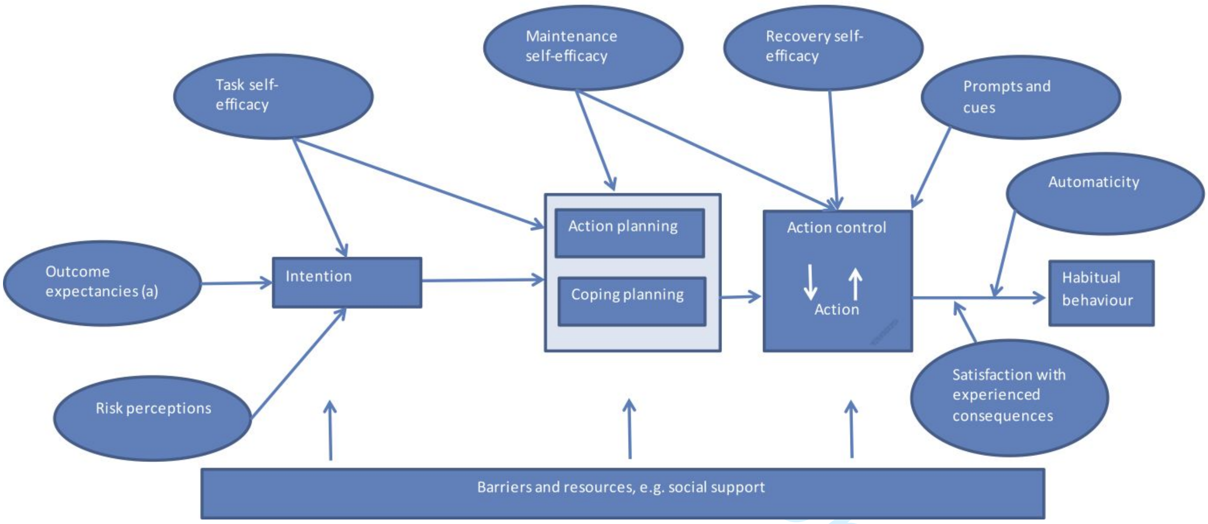

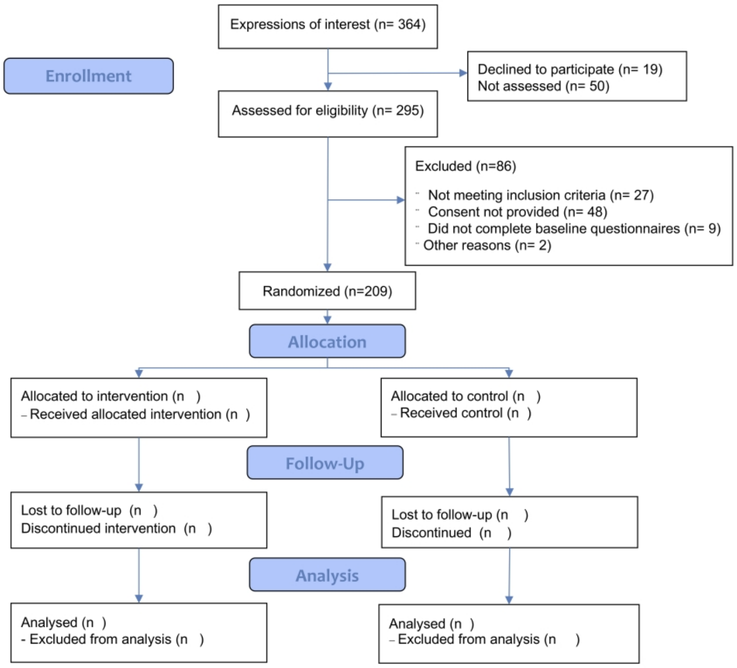

Figure 1 Trial Profile

Supplementary Appendix

This appendix has been provided by the authors to give readers additional information about their work.

Supplement to: Supporting people with type 2 diabetes in effective use of their medicine through a system comprising mobile health technology integrated with clinical care compared with usual care (SuMMiT-D Feasibility): a randomised feasibility trial protocol

For peer review only

Appendix 1

Members of the SuMMiT-D Feasibility Trial Collaborative Group:

Writing Group

A. J. Farmer[1], J.Allen[1], Y.K. Bartlett[2], P. Bower[2], Y. Chi[1], D.P. French[2], B Gudgin[3], E. Holmes[4], R. Horne[5], D.A. Hughes[4], C. Kenning[2], L. Locock[6], J. McSharry[7], L. Miles[2], N. Newhouse[1], R. Rea[1], E. Riga[1], L. Tarassenko[1], C. Velardo[1], N. Williams[1], V. Williams[1], L-M Yu[1].

Author affiliations:
1. University of Oxford, Oxford UK.
2. University of Manchester, Manchester UK.
3. Patient Representative, Oxfordshire, UK.
4. Bangor University, Bangor UK.
5. University College London, UK.
6. University of Aberdeen, Aberdeen UK
7. National University of Ireland, Galway, Eire.

Programme Steering Committee

Independent Members

Chair: K. Khunti; C. Weir, F. Sniehotta, S. England

Investigators

A. Farmer; D.P. French, L-M. Yu

Observers

T. Hutchinson (Funder), E. Chick (Sponsor), C. Velardo, E. Riga, S. M. Robinson

Trial Management Team

J. Allen, Senior Trial Manager; E. Riga, Trial Manager; S. M. Robinson, Trial Coordinator; C. Kenning, Regional Coordinator

Statistics

L-M. Yu, Lead Trial Statistician; N. Williams, Senior Statistician; M. Shanyinde, Statistician

Data Management

S. Kelly, Senior Data Manager; L. Engonidou, Data Manager

IT, Programming and Engineering

For peer review only

D. Judge, Senior IT and Programming Manager; C. Velardo, Senior Research Fellow; Y. Chi, Post-doctoral Research Assistant.

Research Nurses

H. Rutter, Senior Research Nurse; S. Squires, Research Nurse; B. Mundy, Research Nurse; K. Madronal, Research Nurse; I. Noel, Research Nurse; B. I'Anson, Research Nurse; P. Whitbread, Research Nurse

PPI Members

Chair: B. Gudgin; G. Moultrie, M. Simpson, P. Baller, R. Dawett, D. Akker, M. Mistry, S. Mulholland, J. Nugent, G. Sides, D. Parkinson, S. McPhillips

Acknowledgements
In addition to the team members acknowledged above, we would also like to acknowledge the help of the following clinical research networks (CRNs) and participating GP practices supporting recruitment:

**Practice Name**
NIHR CRN: Thames Valley & South Midlands
South Oxford Health Centre
White Horse Medical Practice
Botley Medical Centre
The Rycote Practice
Milman Road Health Centre/Milman Medical
Broadshires Health Centre
Bicester Health Centre
NIHR CRN: West Midlands
Eve Hill Medical Practice
Spring Gardens Group Medical Practice
Burbury Medical Centre
NIHR CRN: South West Peninsula
Brunel Medical Practice
Newquay Health Centre
Glastonbury Surgery
NIHR CRN: Greater Manchester
Block Lane Surgery
Ashcroft Surgery
West Timperley Medical Centre
Chorlton Family Practice
Washway Road Medical Centre
Market Street Medical Practice

Trial Sponsor: University of Oxford. Contact Clinical Trials and Research Governance, Churchill Hospital, Oxford, UK.

For peer review only

Appendix 2.

Supplementary Figure 1

Logic model

Title: Logic model showing anticipated mechanisms of action in the SuMMIT-D Feasibility study, based on the Health Action Process Approach

(a) *Footnote*: (a) This construct includes both beliefs about the necessity of taking medication, and concerns about taking that medication.

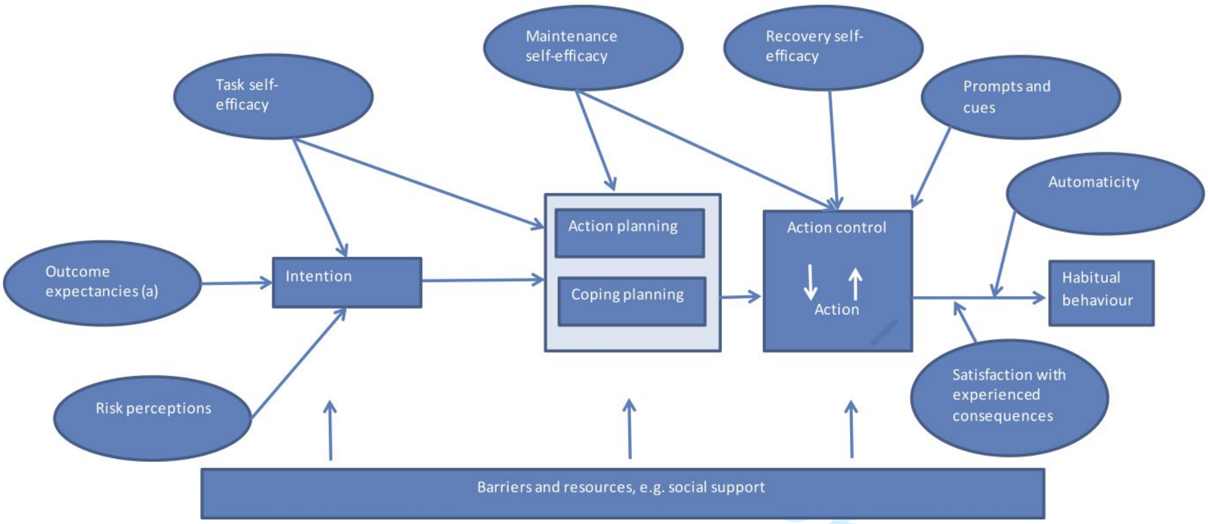

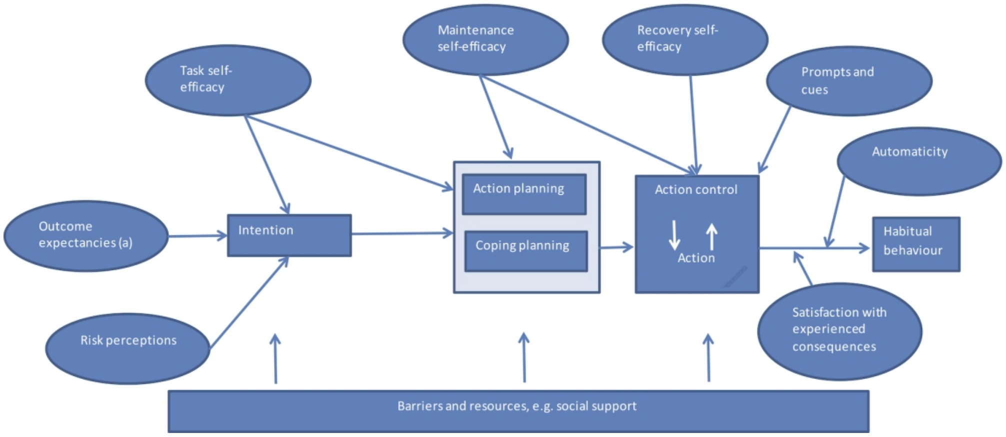

Supplementary Figure 1: Logic Model

Reporting checklist for protocol of a clinical trial.

Based on the SPIRIT guidelines.

Instructions to authors

Complete this checklist by entering the page numbers from your manuscript where readers will find each of the items listed below.

Your article may not currently address all the items on the checklist. Please modify your text to include the missing information. If you are certain that an item does not apply, please write "n/a" and provide a short explanation.

Upload your completed checklist as an extra file when you submit to a journal.

In your methods section, say that you used the SPIRITreporting guidelines, and cite them as:

Chan A-W, Tetzlaff JM, Altman DG, Laupacis A, Gøtzsche PC, Krleža-Jerić K, Hróbjartsson A, Mann H, Dickersin K, Berlin J, Doré C, Parulekar W, Summerskill W, Groves T, Schulz K, Sox H, Rockhold FW, Rennie D, Moher D. SPIRIT 2013 Statement: Defining standard protocol items for clinical trials. Ann Intern Med. 2013;158(3):200-207

		Reporting Item	Page Number
**Administrative information**			
Title	#1	Descriptive title identifying the study design, population, interventions, and, if applicable, trial acronym	1
Trial registration	#2a	Trial identifier and registry name. If not yet registered, name of intended registry	2
Trial registration: data set	#2b	All items from the World Health Organization Trial Registration Data Set	2
Protocol version	#3	Date and version identifier	1
Funding	#4	Sources and types of financial, material, and other support	14
Roles and responsibilities: contributorship	#5a	Names, affiliations, and roles of protocol contributors	13 and 20-21

For peer review only

Roles and responsibilities: sponsor contact information	#5b	Name and contact information for the trial sponsor	12 and 21
Roles and responsibilities: sponsor and funder	#5c	Role of study sponsor and funders, if any, in study design; collection, management, analysis, and interpretation of data; writing of the report; and the decision to submit the report for publication, including whether they will have ultimate authority over any of these activities	12 and 20
Roles and responsibilities: committees	#5d	Composition, roles, and responsibilities of the coordinating centre, steering committee, endpoint adjudication committee, data management team, and other individuals or groups overseeing the trial, if applicable (see Item 21a for data monitoring committee)	20
**Introduction**			
Background and rationale	#6a	Description of research question and justification for undertaking the trial, including summary of relevant studies (published and unpublished) examining benefits and harms for each intervention	4
Background and rationale: choice of comparators	#6b	Explanation for choice of comparators	4/5
Objectives	#7	Specific objectives or hypotheses	5
Trial design	#8	Description of trial design including type of trial (eg, parallel group, crossover, factorial, single group), allocation ratio, and framework (eg, superiority, equivalence, non-inferiority, exploratory)	6
**Methods: Participants, interventions, and outcomes**			
Study setting	#9	Description of study settings (eg, community clinic, academic hospital) and list of countries where data will be collected. Reference to where list of study sites can be obtained	6 and 21

For peer review only

Eligibility criteria	#10	Inclusion and exclusion criteria for participants. If applicable, eligibility criteria for study centres and individuals who will perform the interventions (eg, surgeons, psychotherapists)	8/9
Interventions: description	#11a	Interventions for each group with sufficient detail to allow replication, including how and when they will be administered	6/7 plus TiDierR checklist
Interventions: modifications	#11b	Criteria for discontinuing or modifying allocated interventions for a given trial participant (eg, drug dose change in response to harms, participant request, or improving / worsening disease)	9
Interventions: adherance	#11c	Strategies to improve adherence to intervention protocols, and any procedures for monitoring adherence (eg, drug tablet return; laboratory tests)	TiDIER
Interventions: concomitant care	#11d	Relevant concomitant care and interventions that are permitted or prohibited during the trial	N/A
Outcomes	#12	Primary, secondary, and other outcomes, including the specific measurement variable (eg, systolic blood pressure), analysis metric (eg, change from baseline, final value, time to event), method of aggregation (eg, median, proportion), and time point for each outcome. Explanation of the clinical relevance of chosen efficacy and harm outcomes is strongly recommended	7
Participant timeline	#13	Time schedule of enrolment, interventions (including any run-ins and washouts), assessments, and visits for participants. A schematic diagram is highly recommended (see Figure)	6
Sample size	#14	Estimated number of participants needed to achieve study objectives and how it was determined, including clinical and statistical assumptions supporting any sample size calculations	10
Recruitment	#15	Strategies for achieving adequate participant enrolment to reach target sample size	8

For peer review only

**Methods: Assignment of interventions (for controlled trials)**			
Allocation: sequence generation	#16a	Method of generating the allocation sequence (eg, computer-generated random numbers), and list of any factors for stratification. To reduce predictability of a random sequence, details of any planned restriction (eg, blocking) should be provided in a separate document that is unavailable to those who enrol participants or assign interventions	9
Allocation concealment mechanism	#16b	Mechanism of implementing the allocation sequence (eg, central telephone; sequentially numbered, opaque, sealed envelopes), describing any steps to conceal the sequence until interventions are assigned	9
Allocation: implementation	#16c	Who will generate the allocation sequence, who will enrol participants, and who will assign participants to interventions	9
Blinding (masking)	#17a	Who will be blinded after assignment to interventions (eg, trial participants, care providers, outcome assessors, data analysts), and how	9
Blinding (masking): emergency unblinding	#17b	If blinded, circumstances under which unblinding is permissible, and procedure for revealing a participant's allocated intervention during the trial	9
**Methods: Data collection, management, and analysis**			
Data collection plan	#18a	Plans for assessment and collection of outcome, baseline, and other trial data, including any related processes to promote data quality (eg, duplicate measurements, training of assessors) and a description of study instruments (eg, questionnaires, laboratory tests) along with their reliability and validity, if known. Reference to where data collection forms can be found, if not in the protocol	9

For peer review only

Data collection plan: retention	#18b	Plans to promote participant retention and complete follow-up, including list of any outcome data to be collected for participants who discontinue or deviate from intervention protocols	9
Data management	#19	Plans for data entry, coding, security, and storage, including any related processes to promote data quality (eg, double data entry; range checks for data values). Reference to where details of data management procedures can be found, if not in the protocol	9/12
Statistics: outcomes	#20a	Statistical methods for analysing primary and secondary outcomes. Reference to where other details of the statistical analysis plan can be found, if not in the protocol	10
Statistics: additional analyses	#20b	Methods for any additional analyses (eg, subgroup and adjusted analyses)	10
Statistics: analysis population and missing data	#20c	Definition of analysis population relating to protocol non-adherence (eg, as randomised analysis), and any statistical methods to handle missing data (eg, multiple imputation)	10
**Methods: Monitoring**			
Data monitoring: formal committee	#21a	Composition of data monitoring committee (DMC); summary of its role and reporting structure; statement of whether it is independent from the sponsor and competing interests; and reference to where further details about its charter can be found, if not in the protocol. Alternatively, an explanation of why a DMC is not needed	N/
Data monitoring: interim analysis	#21b	Description of any interim analyses and stopping guidelines, including who will have access to these interim results and make the final decision to terminate the trial	N/A
Harms	#22	Plans for collecting, assessing, reporting, and managing solicited and spontaneously reported adverse events and other unintended effects of trial interventions or trial conduct	10

For peer review only

Auditing	#23	Frequency and procedures for auditing trial conduct, if any, and whether the process will be independent from investigators and the sponsor	N/A
**Ethics and dissemination**			
Research ethics approval	#24	Plans for seeking research ethics committee / institutional review board (REC / IRB) approval	12
Protocol amendments	#25	Plans for communicating important protocol modifications (eg, changes to eligibility criteria, outcomes, analyses) to relevant parties (eg, investigators, REC / IRBs, trial participants, trial registries, journals, regulators)	N/A
Consent or assent	#26a	Who will obtain informed consent or assent from potential trial participants or authorised surrogates, and how (see Item 32)	9
Consent or assent: ancillary studies	#26b	Additional consent provisions for collection and use of participant data and biological specimens in ancillary studies, if applicable	N/A
Confidentiality	#27	How personal information about potential and enrolled participants will be collected, shared, and maintained in order to protect confidentiality before, during, and after the trial	12
Declaration of interests	#28	Financial and other competing interests for principal investigators for the overall trial and each study site	14
Data access	#29	Statement of who will have access to the final trial dataset, and disclosure of contractual agreements that limit such access for investigators	14
Ancillary and post trial care	#30	Provisions, if any, for ancillary and post-trial care, and for compensation to those who suffer harm from trial participation	N/A
Dissemination policy: trial results	#31a	Plans for investigators and sponsor to communicate trial results to participants, healthcare professionals, the public, and other relevant groups (eg, via publication,	14

For peer review only

		reporting in results databases, or other data sharing arrangements), including any publication restrictions	
Dissemination policy: authorship	#31b	Authorship eligibility guidelines and any intended use of professional writers	N/A
Dissemination policy: reproducible research	#31c	Plans, if any, for granting public access to the full protocol, participant-level dataset, and statistical code	14
**Appendices**			
Informed consent materials	#32	Model consent form and other related documentation given to participants and authorised surrogates	Appendix
Biological specimens	#33	Plans for collection, laboratory evaluation, and storage of biological specimens for genetic or molecular analysis in the current trial and for future use in ancillary studies, if applicable	N/A

None The SPIRIT checklist is distributed under the terms of the Creative Commons Attribution License CC-BY-ND 3.0. This checklist can be completed online using https://www.goodreports.org/, a tool made by the EQUATOR Network in collaboration with Penelope.ai

For peer review only

<table>
<tr><th colspan="4">TIDieR checklist for the Supporting people with type 2 diabetes in effective use of their medicine through mobile health technology integrated with clinical care (SuMMiT-D) feasibility trial</th></tr>
<tr><td>1.</td><td>Brief name</td><td colspan="2">Supporting people with type 2 diabetes in effective use of their medicine through mobile health technology integrated with clinical care (SuMMiT-D Feasibility)</td></tr>
<tr><td rowspan="3">2.</td><td rowspan="3">Rationale or theory</td><td colspan="2">Systematic reviews of text messages to support patients to adhere to treatment, and of mobile health interventions in diabetes, identify some effective interventions. There are a few trials testing the impact of brief messaging in type 2 diabetes, but they do not have systematically developed interventions based on theory and evidence and are at risk of bias.[1,2] Recent trials of text-messaging for cardiovascular risk prevention and blood pressure lowering have shown clinically relevant changes in outcomes compared with usual care.[3,4]
In addition, there is substantial evidence[5] that tailored interventions are more effective than generic interventions. Tailored interventions may be seen by recipients as more personally relevant, so they will be more likely to attend to, read, understand, and act on them. In addition, tailored interventions are designed to change determinants of the target behaviour that are relevant to particular individuals or to small subgroups of individuals; they therefore more precisely target the determinants of the individual's behaviour.
SuMMiT-D (SUpport through Mobile Messaging and digital health Technology for Diabetes) is a programme of work comprised of three phases: formative work, a feasibility trial and a large scale, effectiveness randomised controlled trial of a mobile phone-based system intended to deliver brief, tailored, behaviour-change messages to people with type 2 diabetes focusing on use of medication, lifestyle and other aspects of diabetes management. In the formative work for this trial, we identified theoretical constructs and features of intervention content found to be associated with medication adherence in patients with type 2 diabetes and mapped these onto a standard taxonomy for behaviour change techniques (BCTs), that is, active ingredients of interventions used to promote behavioural change[6,7]. We then developed a large set of messages to target each BCT, and examined which types of messages are most useful and easy to understand for people starting and taking an oral diabetes medicine, and the extent to which it might be helpful for patients to decide on the types of messages they want to receive. We received input from approximately 300 patients with type 2 diabetes and healthcare professionals caring for this patient population. Preliminary findings of this trial development work were used to develop this feasibility trial with scope for further refinement of the system for the main clinical trial.</td></tr>
<tr><td>Usual care and one non-health-related message a month</td><td>Condition-specific tailored text messaging system and usual care</td></tr>
<tr><td>Active control: Infrequent non-health related messages sent to all participants,

The purpose of the infrequent messages is to:
i) Maintain participant interest in the trial.
ii) Make it less clear who was getting which intervention</td><td>i. Participants will be sent up to four automated text-messages per week with an average frequency of three per week relating to diabetes management and use of medicine.
ii. The library of text-messages uses different behaviour change techniques to target health-related behaviour change relating to use of medicines, as well as messages targeting other aspects of diabetes care (including diet and exercise)..</td></tr>
</table>

For peer review only - http://bmjopen.bmj.com/site/about/guidelines.xhtml

		iii) Exclude receipt of "any SMS" as effecting health-related behaviour	iii. Frequency of messages received using a particular type of behaviour change technique can be modified based on a participant's response to individual messages received.<br>The style of messages will be patient-centred and will encourage patients to seek further relevant information (including the use of links where possible to selected external websites e.g. Diabetes UK).
**3.**	**Materials**	Available health materials on type 2 diabetes routinely provided by the health care service. All participants were sent a system user guide by email.	
**4.**	**Procedures**	1. Language and timing of messages selected by participant<br>2. Types of brief messages<br>a) Welcome message (confirming sign-up)<br>b) Message with options for changing delivery time<br>c) Non-health related messages sent every 4 weeks for 6-months<br>d) Thank you message at end of study	1. Timing of messages selected by participant<br>2. Types of brief messages<br>a) Welcome messages (confirming sign-up)<br>b) Message with instructions about use of system<br>c) Brief treatment and lifestyle adherence support messages randomly selected from library (with rules that ensured individual messages were not repeated) and sent several times per week, each week for 6-months<br>d) Reminders of how to use the system to "Like" and "Dislike" messages, STOP and PAUSE sending of messages<br>e) Thank you message at end of study
**5.**	**Intervention provider**	Automated SMS text-message delivery platform using open-source software and third party bulk SMS-delivery provider	
**6.**	**Modes of delivery**	Intervention delivered via 160 characters SMS-text sent to individual participant's own handset. Initial message sent to all enrolled participants is Welcome message confirming sign-up, thereafter after automated messages sent to individual participants as per randomised allocation for 6-months.	
**7.**	**Location where intervention occurred**	Community	
**8.**	**Number of times intervention was delivered over what time period**	Brief trial related and non-health messages sent once every 4 weeks for 6-months	Brief messages sent three (min) to four (max) times per week for 6-months
**9.**	**What, why, when, how intervention was personalised or adapted**	1. Timing of messages selected by participant.	1. Timing of messages selected by participant<br>2. Linkage of the proportion of actual vs. expected proportion of prescribed medication to tailor a small number of text-messages

For peer review only

**10.**	**Modifications during the trial**	Nil	Nil
**11.**	**Planned intervention delivery**	Brief messages will be sent using an automated system independent of trial and clinical staff. Participants will be told that not everyone will be receiving the exact same messages. Participants will also be asked not to share their health messages with others. Intervention fidelity (receipt of messages and linkage to an individual) will be checked by response to the REGISTER request. Message delivery reports will be monitored throughout the trial to check the intervention is being delivered as planned. Messages not delivered (network unavailable etc.) will be resent up to three times. Where message delivery fails after three sequential attempts a failed message protocol will be initiated to track the participant and up-date their phone number if required.	
**12.**	**Actual intervention delivery**	Will be monitored during trial, blind to allocated group	

1. Ricci-Cabello I, Bobrow K, Islam SMS, Chow CK, Maddison R, Whittaker R, et al. Examining Development Processes for Text Messaging Interventions to Prevent Cardiovascular Disease: Systematic Literature Review. JMIR mHealth and uHealth. 2019 Mar 29;7(3):e12191.
2. Farmer AJ, McSharry J, Rowbotham S, McGowan L, Ricci-Cabello I, French DP. Effects of interventions promoting monitoring of medication use and brief messaging on medication adherence for people with Type 2 diabetes: a systematic review of randomized trials. Diabet Med. 2015 Oct 15;33(5):565–79.
3. Chow CK, Redfern J, Hillis GS, Thakkar J, Santo K, Hackett ML, et al. Effect of Lifestyle-Focused Text Messaging on Risk Factor Modification in Patients With Coronary Heart Disease: A Randomized Clinical Trial. JAMA : the journal of the American Medical Association. American Medical Association; 2015 Sep 22;314(12):1255–63.
4. Bobrow K, Farmer AJ, Springer D, Shanyinde M, Yu L-M, Brennan T, et al. Mobile Phone Text Messages to Support Treatment Adherence in Adults With High Blood Pressure (SMS-Text Adherence Support [StAR]): A Single-Blind, Randomized Trial. Circulation. 2016 Feb 9;133(6):592–600.
5. Kassavou A, Sutton S. Automated telecommunication interventions to promote adherence to cardio-metabolic medications: meta-analysis of effectiveness and meta-regression of behaviour change techniques. Health Psychology Review. Taylor & Francis; 2017 Sep 12;12(1):25–42.
6. Michie S, Richardson M, Johnston M, Abraham C, Francis J, Hardeman W, et al. The Behavior Change Technique Taxonomy (v1) of 93 Hierarchically Clustered Techniques: Building an International Consensus for the Reporting of Behavior Change Interventions. Ann Behav Med. 2013 Mar 20;46(1):81–95.
7. Long H, Bartlett YK, Farmer AJ, French DP. Identifying Brief Message Content for Interventions Delivered via Mobile Devices to Improve Medication Adherence in People With Type 2 Diabetes Mellitus: A Rapid Systematic Review. J Med Internet Res. 2019;21(1):e10421–19.

For peer review only - http://bmjopen.bmj.com/site/about/guidelines.xhtml
For peer review only

BMJ Open

Supporting people with type 2 diabetes in effective use of their medicine through mobile health technology integrated with clinical care (SuMMiT-D Feasibility): a randomised feasibility trial protocol

Journal:	*BMJ Open*
Manuscript ID	bmjopen-2019-033504.R1
Article Type:	Protocol
Date Submitted by the Author:	30-Oct-2019

Complete List of Authors:	Farmer, Andrew; University of Oxford, Nuffield Department of Primary Care Health Sciences<br>Allen, Julie; University of Oxford, Nuffield Department of Primary Care Health Sciences<br>Bartlett, Kiera; The University of Manchester, The Division of Psychology and Mental Health<br>Bower, Peter; The University of Manchester, Division of Population Health, Health Services Research & Primary Care<br>Chi, Yuan; University of Oxford, The Institute of Biomedical Engineering<br>French, David; The University of Manchester, The Division of Psychology and Mental Health<br>Gudgin, Bernard; Patient Representative<br>Holmes, EA; Bangor University<br>Horne, Robert; University College London, UCL School of Pharmacy<br>Hughes, Dyfrig; Bangor University, Centre for Health Economics and Medicines Evaluation<br>Kenning, Cassandra; The University of Manchester, Institute of Population Health<br>Locock, Louise; University of Aberdeen, Health Service Research<br>McSharry, Jenny; National University of Ireland Galway, Health Behaviour Change Research Group, School of Psychology<br>Miles, Lisa; The University of Manchester, Division of Psychology and Mental Health<br>Newhouse, Nikki; University of Oxford, Nuffield Department of Primary Care Health Sciences<br>Rea, Rustam ; University of Oxford, Nuffield Department of Primary Care Health Sciences<br>Riga, Evgenia; University of Oxford, Nuffield Department of Primary Care Health Sciences<br>Tarassenko, Lionel; University of Oxford, Institute of Biomedical Engineering<br>Velardo, Carmelo; University of Oxford, Institute of Biomedical Engineering<br>Williams, Nicola; University of Oxford, Nuffield Department of Primary Care Health Sciences<br>Williams, Veronika; University of Oxford, Nuffield Department of Primary Care Health Sciences<br>Yu, Ly-Mee; University of Oxford, Nuffield Department of Primary Care

	Health Sciences
**Primary Subject Heading**:	Diabetes and endocrinology
Secondary Subject Heading:	General practice / Family practice, Communication, Health services research
Keywords:	type 2 diabetes, digital health, behaviour change intervention, PRIMARY CARE, randomised controlled trial, process evaluation

SCHOLARONE™
Manuscripts

For submission to BMJ Open

Supporting people with type 2 diabetes in effective use of their medicine through mobile health technology integrated with clinical care (SuMMiT-D Feasibility): a randomised feasibility trial protocol

The SuMMiT-D Collaborative Group

Andrew J. Farmer[1], Julie Allen[1], Y.Kiera Bartlett[2], Peter Bower[2], Yuan Chi[1], David P. French[2], Bernard Gudgin[3], Emily Holmes[4], Rob Horne[5], Dyfrig A. Hughes[4], Cassandra Kenning[2], Louise Locock[6], Jennifer McSharry[7], Lisa Miles[2], Nikki Newhouse[1], Rustam Rea[1], Evgenia Riga[1], Lionel Tarassenko[1], Carmelo Velardo[1], Nicola Williams[1], Veronika Williams[1], Ly-Mee Yu[1].

Author affiliations:
1. University of Oxford, Oxford UK.
2. University of Manchester, Manchester UK.
3. Patient Representative, Oxfordshire, UK.
4. Bangor University, Bangor UK.
5. University College London, UK.
6. University of Aberdeen, Aberdeen UK
7. National University of Ireland, Galway, Eire.

Abstract: 300
Main Text: 3805/4,000

Figures: 2
Tables: 2

Date and version No: 09-DEC-2019; v0.66

Correspondence to: Andrew Farmer, Nuffield Department of Primary Care Health Sciences, Woodstock Road, Oxford OX2 6GG. andrew.farmer@phc.ox.ac.uk
Tel: +44 1865 289280

Key Words
Type 2 diabetes, digital health, behavioural change intervention, primary care, feasibility study, process evaluation, randomized controlled trial.

Abstract

Introduction Type 2 diabetes is common, affecting over 400 million people worldwide. Risk of serious complications can be reduced through use of effective treatments and active self-management. However, people are often concerned about starting new medicines and face difficulties in taking them regularly. Use of brief messages to provide education and support self-management, delivered through mobile phone-based text-messages can be an effective tool for some long-term conditions. We have developed messages aiming to support patients' self-management of type 2 diabetes in the use of medications and other aspects of self-management, underpinned by theory and evidence. The aim of this trial is to determine the feasibility of a large-scale clinical trial to test the effectiveness and cost-effectiveness of the intervention, compared to usual care.

Methods and analysis The feasibility trial will be a multi-centre individually randomised, controlled trial in primary care recruiting adults (≥35 years) with type 2 diabetes in England. Consenting participants will be randomised to receive short text-messages three times a week with messages designed to produce change in medication adherence or non-health related messages for six months. The aims are to test recruitment methods, retention to the study, the feasibility of data collection and the mobile-phone and web-based processes of a proposed definitive trial and to refine the text messaging intervention. The primary outcome is the rate of recruitment to randomisation of participants to the trial. Data, including patient reported measures, will be collected online at baseline and the end of the six-month follow-up period. With 200 participants (100 in each group), this trial is powered to estimate 80% follow up within 95% confidence intervals of 73.8% to 85.3%. The analysis will follow a pre-specified plan.

Ethics and Dissemination Ethics approval was obtained from the West of Scotland Research Ethics Committee 05. The results will be disseminated through conference presentations, peer-reviewed journals and will be published on the trial website: www.summit-d.org

SuMMiT-D (SUpport through Mobile Messaging and digital health Technology for Diabetes)

Trial Registration
ISRCTN13404264; date of registration 10 October 2018

Article Summary

Strengths and limitations of this study

1. The use of brief messages to support self-management and provide motivation and education, delivered through mobile phone-based text-messages can be an effective tool for some long-term conditions.
2. There are a few trials testing the impact of brief messaging in type 2 diabetes; although some have systematically-developed messages mapped to theoretical constructs, many are at risk of bias and may be limited in their application to specific contexts.
3. The objective of the SuMMiT-D Feasibility trial is to test recruitment and randomisation of participants, and collection of proposed primary and secondary outcome data in planning for a large, outcomes based, randomised controlled trial.
4. The messages evaluated in this study are (i) targeted at constructs identified as being related to medication adherence by systematic reviews of the literature, and (ii) demonstrated to have fidelity to intended behaviour change techniques (as rated by experts) and acceptability to target population (as rated by patients)
5. Potential mechanisms for the action of the brief messages will be assessed through changes in constructs that are related to medication-taking behaviour, questions based on the Technology Acceptance model, and self-report medication adherence.

Introduction

Type 2 diabetes is one of the most common long-term conditions affecting 422 million people worldwide[1] and 4.7 million people in the UK.[2] It can lead to major complications including cardiovascular disease, renal failure and neuropathy.[3] The global burden of diabetes is projected to reach up to 2.2% of global gross domestic product,[4] and many of these costs are due to preventable complications. Prevention includes the use of treatments of proven efficacy[5,6] alongside supporting self-management.[7] However, concerns about medicines and difficulties in taking them regularly, whether intentional or unintentional, are common[8] and lead to poor diabetes control and increased use of health resources.[9] The cost of non-adherence to diabetes medication in the United Kingdom has been estimated at £100 million per year in avoidable treatment costs.[10] A wide range of services are available to support people in better use of their medicines, but evidence of their effectiveness and cost-effectiveness is weak. [11] These services are often targeted at particular groups of individuals and are frequently designed as "one-off" interventions. Examples include medication reviews, education and addressing cost issues. [12] Understanding and improving this situation could make a major contribution to health and health-care costs.

Systematic reviews do not provide strong evidence to support the wider use of current approaches to developing interventions to improve adherence.[13] Brief messages delivered at a wide scale and low cost via digital health systems added to usual care have been shown to be effective in improving health for some conditions and are a promising approach to the problem.[14]

Systematic reviews of text messages to support patients to adhere to treatment, and of mobile health interventions in diabetes, identify some effective interventions. There are a few trials testing the impact of brief messaging in type 2 diabetes; although some have systematically-developed messages mapped to theoretical constructs, many are at risk of bias and may be limited in their application to specific contexts.[14,15] Recent trials of text-messaging for cardiovascular risk prevention and blood pressure lowering have shown clinically relevant changes in outcomes compared with usual care.[16,17]

In addition, there is substantial evidence that tailored interventions are more effective than generic interventions.[18] Tailored interventions may be seen by

For peer review only

recipients as more personally relevant, so they will be more likely to attend to, read, understand, and act on them. In addition, tailored interventions are designed to change determinants of the target behaviour that are relevant to particular individuals or to small subgroups of individuals; they therefore more precisely target the determinants of the individual's behaviour.

SuMMiT-D (SUpport through Mobile Messaging and digital health Technology for Diabetes) is a programme of work comprised of three phases: formative work, a feasibility trial and a large scale, effectiveness randomised controlled trial of a mobile phone-based system intended to deliver brief, tailored, behaviour-change messages to people with type 2 diabetes focusing on use of medication. The intervention is intended to focus on a broad range of individuals with type 2 diabetes, but those with younger onset diabetes and using insulin alone were not included, as these features can require care using different pathways. In the formative work for this trial, we identified theoretical constructs and features of intervention content found to be associated with medication adherence in patients with type 2 diabetes and mapped these onto a standard taxonomy for behaviour change techniques (BCTs), that is, active ingredients of interventions used to promote behavioural change.[19,20]. We then developed a large set of messages to target each BCT, and examined which types of messages are most useful and easy to understand for people starting and taking an oral diabetes medicine, and the extent to which it might be helpful for patients to decide on the types of messages they want to receive. We received input from approximately 300 patients with type 2 diabetes and healthcare professionals caring for this patient population. Preliminary findings of this trial development work were used to develop this feasibility trial with scope for further refinement of the system for the main clinical trial.

The primary objective of the SuMMiT-D Feasibility trial is to test recruitment and randomisation of participants to the trial. We will test collection of planned primary and secondary outcome data in planning for a large, outcomes-based, randomised controlled trial. We will assess the feasibility and acceptability of the intervention for patients and healthcare professionals; the willingness of participants to be randomised; follow-up rates; resource use and trial procedures. We will also carry out a process evaluation of how the system is used and refine the way it is embedded within usual care. Potential mechanisms for the action of the brief messages will be assessed through changes in hypothesised health psychology

For peer review only - http://bmjopen.bmj.com/site/about/guidelines.xhtml

constructs relating to use of medication, self-report medication adherence,[21] and questions based on the Technology Acceptance model.[22]

Methods and analysis

The SuMMiT-D Feasibility trial protocol is reported according to SPIRIT and EMERGE recommendations.[23,24]

Patient and Public Involvement

Patient members of the public are integral to this trial. A panel of eleven patient and public involvement (PPI) members with type 2 diabetes was set up and continues to inform our work, reviewing all patient documentation and research findings, and supports the development of the intervention.

All patient facing documents for the SuMMiT-D feasibility trial, including, the participant information sheet, informed consent form, posters, user guides, and website were reviewed by PPI panel members. The panel has been kept up to date with frequent trial updates in the form of an email newsletter, also published on the trial website (www.summit-d.org). The results of the study will be made available to trial participants, PPI panel members and participating GP practices on the trial website.

Trial Design

SuMMiT-D Feasibility is a primary care based, two-arm, individually randomised controlled, parallel group trial aiming to recruit a total of 200 patients across twenty general practice sites in England. Patients with type 2 diabetes will be randomly allocated to receive an individually tailored Short Messaging System (SMS) text-messaging based intervention for 26 weeks that aims to encourage and support them in developing a habit of taking their medication as intended (that is, to promote effective implementation of dosing and treatment continuation)[25] and provides hints and tips to help them with other aspects of living with the condition alongside usual care (treatment arm), or to usual care with the addition of infrequent non-health related messages (control arm) (Figure 1).

For peer review only

Intervention

Intervention arm: Condition-specific tailored text messaging system and usual care

Participants assigned to the intervention group will receive brief health related SMS text messages, based on systematic review of the evidence identifying determinants of medication taking behaviour.[20] Messages were developed based on systematic review evidence by experts,[20] and were refined in an iterative process of ensuring acceptability based on patient feedback and demonstration of fidelity to intended behaviour change determinants, as rated by an independent group of experts.[26] A more detailed description of the intervention is given in the accompanying TIDieR checklist. Examples of messages are given in the supplementary file (Appendix 1).

The intervention is a digital health system with the following components:

i. Participants will be sent up to four automated text-messages per week with an average frequency of three per week relating to diabetes management and use of medicine.

ii. The library of text-messages uses different behaviour change techniques to target health-related behaviour change relating to use of medicines, as well as messages targeting other aspects of diabetes care (including diet and exercise).

iii. The types of messages sent will be tailored to participants based on time since starting new glucose, blood pressure and cholesterol-lowering medication and current smoking status.

iv. Frequency of messages received using a particular group of behaviour change techniques can be modified based on a participant's response to individual messages received through sending a text-message in response to a particular message. Participants may incur a cost for sending messages in response depending on their network plan.

v. The style of messages will be patient-centred and will encourage patients to seek further relevant information (including the use of links where possible to selected external websites e.g. Diabetes UK).

Control arm: Usual care and one non-health-related message a month

Participants assigned to usual care will receive non-health related text-messages at a frequency of approximately one every four weeks. Care will otherwise not differ from usual care.

Outcomes

The primary outcome is the rate of recruitment to randomisation of participants to the trial. Details of outcomes and measurement are shown in Table 1. We will measure

For peer review only - http://bmjopen.bmj.com/site/about/guidelines.xhtml

recruitment against planned recruitment rates for the proposed main trial and number of people showing an interest in the trial and not proceeding or those who withdraw from the control group and give a reason.

Secondary outcomes (Table 1) focus on feasibility of collection of clinical and economic measurement data for the proposed main trial. They include the availability of HbA_{1c}, systolic blood pressure and total to HDL cholesterol ratio data from medical records, retention rates, and proportion of prescribing data available. A self-report questionnaire will assess: medication adherence,[21] health status with the EQ-5D-5L,[27] resource use for the health economic analysis, technology acceptance,[22] and constructs targeted by SMS and hypothesised to mediate the effects of the intervention on adherence.[28]

Procedures and assessments

Participants who express interest in taking part in the trial will be screened by the trial team and will consent and submit their baseline questionnaires either online or on paper according to their preference. Participants will be randomised by the trial team and will receive messages for 26 weeks from randomisation to final follow-up. All participants will be asked to complete the same set of questionnaires at baseline and at the end of their 26-week follow-up period. Medical notes reviews will be conducted at baseline, six- and 18-months from randomisation.

Recruitment

Potential participants will be identified through general practices in England. Participating practices fall under four Clinical Research Networks (CRNs): Thames Valley & South Midlands, Greater Manchester, West Midlands and South West Peninsula.

Healthcare professionals will screen their type 2 diabetes clinic lists and will invite eligible patients. Patients will receive an invitation letter and summary information leaflet. Eligible patients may also be contacted by phone, email or text by the practice team up to three times.

Expressions of interest

People interested in taking part can send their full name by SMS text-message to the trial team to register their interest. If potential participants have any difficulties in

For peer review only

registering their interest in the trial, they will also be able to contact the team via phone or email and will receive support in registering as required.

Screening assessment

Following an expression of interest, further information about the study will be provided by email or by post as requested. Eligibility will be confirmed by phone.

Inclusion criteria

Eligible participants are ≥35 years of age, taking oral glucose lowering treatment, blood pressure lowering treatment or lipid-lowering treatment either alone or in combination. They have access to a mobile phone and are able, if necessary, with help (e.g. relative, friend, neighbour), to send, understand and retrieve brief SMS text-messages in the English language. Participants who are using insulin treatment without also concomitant use of oral glucose lowering treatment; who are pregnant, within three months post-partum or planning pregnancy during the course of the trial; have a serious medical condition that, in the opinion of the investigator, makes them ineligible; have been admitted to hospital within the last three months for hyper- or hypoglycaemia, are ineligible.

Informed consent

Participants will provide consent either on-line or on paper.

Baseline and follow up assessments

The following questionnaires will be administered (online or by post) at the baseline assessment: the Medication Adherence Report Scale (MARS) self-report scale,[21] the EuroQol 5-Dimension, 5-Level (EQ-5D-5L) scale,[27] a set of measures developed for the study based on the technology acceptance model,[22] and a healthcare utilisation record to allow healthcare resource use to be costed. A further set of measures assess the constructs that will be targeted by the BCTs within the messages. These constructs are hypothesised to mediate the effects of the intervention on medication taking behaviour, in line with the logic model we have developed based on the Health Action Process Approach (Figure 2, Table 1 and Appendix 2).[28]

All self-report data will be collected either online or on paper according to the participant’s preference. The same measures are collected at 26 weeks after randomisation alongside clinical record data.

For peer review only - http://bmjopen.bmj.com/site/about/guidelines.xhtml

Randomisation

Participants will be randomised after consent and when all baseline assessments have been completed. Participants will be allocated in a 1:1 ratio to either the intervention or the control arm. Randomisation will be done using a validated secure web-based randomisation programme (Sortition) provided by the University of Oxford Primary Care Clinical Trials Unit (PC-CTU). Allocation will be carried out with a non-deterministic minimisation algorithm to ensure groups are balanced for important baseline prognostic and other factors: study site, age (<65/≥65 years), gender (male/female), duration of diabetes (<5 years/≥5 years), number of medications (<5/≥5). The allocated intervention will be implemented directly by the platform on which the digital health system is run. Apart from the qualitative research team and the engineering team, allocation is blinded to all other trial and healthcare staff. Due to the nature of the study, unblinding is not required during the trial.

Discontinuation of intervention or withdrawal from trial

Participants can withdraw from the trial at any time. Participants can also choose to Pause or Stop receipt of text messages by sending a text-message or contacting the trial office by telephone or post. Adverse events are collected, and serious unexpected adverse events related to the intervention are determined by the chief investigator and reported in line with local procedures.

Statistical Analysis

Power

With 200 participants (100 in each group) the feasibility trial is powered to estimate 80% follow up within 95% confidence intervals of 73.8% to 85.3%.

Analysis

The primary outcome will be the number of patients recruited to randomisation as a proportion (with 95% confidence interval) of the target recruitment number. Secondary outcomes will be reported overall and separately by allocated arm.

An intention-to-treat approach will be used for an exploratory analysis of secondary outcomes by allocated arm. Continuous outcomes will be analysed using an analysis of covariance adjusting for minimisation factors. Results will be presented as an adjusted difference in means with 95% confidence interval. Binary

For peer review only

outcomes will be analysed in a similar way using log binomial regression models (adjusting for minimisation factors). Results will be presented as relative risks with 95% confidence intervals. There will be no formal assessment of treatment efficacy for this feasibility study.

Analysis of utility and costs

Data collected on healthcare utilisation will be assessed for completeness. The items recorded include patient contacts with healthcare professionals, use of primary care services, hospital outpatient appointments, accident and emergency visits, and inpatient stays, prescribed and purchased medications, and personal social services. The proportion of complete and returned questionnaires will be reported. Total costs associated with each item of resource will be estimated based on unit costs from standard sources,[29,30] and presented descriptively.

The costing is conducted from the perspective of the NHS and therefore the cost to the patient is not considered, we will however, estimate costs of responding to text messages using estimates from average network tariffs and data collected on the quantity of digital information transmitted.

Responses to the EQ-5D-5L questionnaire will also be examined for completeness and reported by proportion of returned questionnaires and items with a valid response. Health utility values corresponding to patients' EQ-5D-5L profiles will be calculated by mapping the 5L descriptive system data onto the 3L value set,[31] and presented descriptively.

Process evaluation

We will perform a mixed-methods process analysis, consisting of a qualitative element and a quantitative element. The qualitative process evaluation will examine the experience of participants and health professionals in their use of and implementation of the messaging system. Longitudinal interviews with up to 30 participants will take prior to use of the system and after the 26-week follow-up. Additionally, up to 30 healthcare professionals will be invited to share their experiences of taking part in this trial.

Interviews with trial participants

Participants who consent to taking part in the embedded qualitative study will be purposefully selected by characteristics including age, gender, length of time of

For peer review only

diabetes and type of medication, and invited to share their views on engagement with and content of the messaging system, and to provide insight into how it was implemented in daily life, identify issues around potential attrition and inform final procedures for the main trial.

Interviews and/or focus groups with healthcare professionals

Healthcare staff from practices taking part in the trial will be invited to take part in focus groups or qualitative interviews to share their experience of recruiting for this trial, and how the intervention was implemented in routine clinical care. Eligible staff will be those with a potential role in the implementation of a text-message system or who have contact with patients with type 2 diabetes. These will include GPs, practice nurses, receptionists and healthcare assistants.

Analysis of qualitative data: trial participants

Thematic analysis will be used inductively to identify key issues, focussing on similarities and differences identified in themes to understand the issues that are important to the participants.[32] We will use NVivo and mind-mapping software to code data, identify themes and interpret these and their relationship. The data analysis will be inductive.

Analysis of qualitative data: healthcare professionals

We will follow a semi-structured topic guide based on the core constructs of extended normalization process theory (NPT):[33] Coherence, Reflexive Monitoring, Cognitive Participation and Collective Action to ensure key areas are covered while allowing flexibility to follow up topics introduced by participants. NVivo software will be used to facilitate data organisation, analysis and the development of themes. The analysis will use a thematic approach informed by NPT.[32]

Quantitative process analysis

To facilitate understanding of mechanism of the intervention, the effect of the intervention on the set of measures based on the logic model (Figure 2 and Table1) will be analysed using a per-protocol analysis for responders, according to allocated group. This will identify if these measures have been affected by the intervention. A further analysis will examine how these measures in the logic model (controlling for baseline) predict change in self-reported medication adherence using hierarchical multiple regression techniques.[34] This analysis will thereby assess extent of association between measures in the logic model and self-reported medication

For peer review only

adherence.[35] These analyses will be used in conjunction with the qualitative analyses described above to inform decisions about selection of SMS messages relating to particular BCTs, and potentially refinement of the logic model and selection of measures for the full trial. Receipt of messages on participants' mobile phones will be monitored throughout the trial.

Data Management

All trial data will be entered on electronic case report forms. The clinical database is built on REDCap (Research Electronic Data Capture), a secure, web-based application designed to support data capture for research studies.[36]

Ethics and dissemination

The trial will be conducted according to the principles of the Declaration of Helsinki and in accordance with other relevant national guidelines, regulations, acts and using Good Clinical Practice guidelines. The University of Oxford sponsors the trial.

The protocol, informed consent form, participant information leaflet and all other patient-facing material have been submitted and approved by the West of Scotland Research Ethics Committee 05 (HRA ref: 18/WS/0173). The role of the Trial Steering Committee is taken on by the NIHR Programme Steering Committee.

Dissemination plan

The results of this trial will be submitted to a peer reviewed journal for publication.

Recruitment to the SuMMiT-D Feasibility Study began with the first participant randomised on 26 November 2018 and the last participant randomised on 16th April 2019. Reporting of the trial is anticipated in the first quarter of 2020.

Discussion

SuMMiT-D Feasibility will inform the design of a future large-scale randomised controlled trial, aiming to estimate the clinical and cost effectiveness of the text-messaging intervention and identify difficulties that might be encountered in practice. If effective and implemented in the NHS, this intervention could help reduce the

For peer review only

burden of complications and increased costs associated with non-adherence. This research could also offer a model for technology-based self-management support that could be extended to other aspects of diabetes care and other long-term conditions.

The SuMMiT-D Collaborative Study Group are listed in Appendix 3 (Supplementary file) with their roles.

Author contributions

AF	A. J. Farmer,	andrew.farmer@phc.ox.ac.uk
JA	J. Allen,	julie.allen@phc.ox.ac.uk
KB	Y.K. Bartlett,	kiera.bartlett@manchester.ac.uk
PB	P. Bower,	peter.bower@manchester.ac.uk
YC	Y. Chi,	yuan.chi@eng.ox.ac.uk
DF	D.P. French,	david.french@manchester.ac.uk
BG	B. Gudgin	bernard_gudgin@compuserve.com
EH	E. Holmes,	e.holmes@bangor.ac.uk
RH	R. Horne	r.horne@ucl.ac.uk
DH	D.A. Hughes	d.a.hughes@bangor.ac.uk
CK	C. Kenning,	cassandra.kenning@manchester.ac.uk
LL	L. Locock,	louise.locock@abdn.ac.uk
JM	J. McSharry,	jenny.mcsharry@nuigalway.ie
LM	L. Miles	lisa.miles-2@manchester.ac.uk
NN	N. Newhouse,	nicola.newhouse@phc.ox.ac.uk
RR	R. Rea	rustam.rea@nhs.net
ER	E. Riga,	jenny.riga@phc.ox.ac.uk
LT	L. Tarassenko,	lionel.tarassenko@eng.ox.ac.uk
CV	C. Velardo,	carmelo.velardo@eng.ox.ac.uk
NW	N. Williams,	nicola.williams@phc.ox.ac.uk
VW	V. Williams,	veronika.williams@phc.ox.ac.uk
LM-Y	L-M Yu.	ly-mee.yu@phc.ox.ac.uk

For peer review only

Each author has contributed significantly to, and is willing to take public responsibility for, one or more aspects of the study. AF, LL and DF conceived the study, AF, DF, PB, LL, RR, VW, LL, JM, CV, LT, DH, RH, LM-Y were involved in planning the study. AF, JA, KB, PB, YC, DF, BG, EH, RH, DH, CK, LL, JM, LM, NN, RR, ER, LT, CV, NW, VW, LM-Y, are involved in carrying out the study. KB, YC, EH, CK, LM, NN and ER are involved in data collection. AF and ER drafted the initial manuscript; AF, JA, KB, PB, YC, DF, BG, EH, RH, DH, CK, LL, JM, LM, NN, RR, ER, LT, CV, NW, VW, LM-Y provided revisions and approved the final version.

Funding This article presents independent research funded by the National Institute for Health Research (NIHR) under its Programme Grants for Applied Research as part of a wider programme of work (RP-PG-1214-20003). The views expressed are those of the authors and not necessarily those of the NHS, the NIHR or the Department of Health and Social Care. The SuMMiT-D research team acknowledge the support of the NIHR through the Clinical Research Networks (AF NIHR Senior Investigator and AF and RR funding from NIHR Oxford Biomedical Research Consortium)

Competing interests All authors have completed the ICMJE uniform disclosure form at www.icmje.org/coi_disclosure.pdf and declare: all authors had financial support from NIHR Programme Grants for Applied Research RP-PG-1214-20003 for the submitted work; no authors reported financial relationships with any organisations that might have an interest in the submitted work in the previous three years; LT reported personal fees from Sensyne Health, CV reported salary support from Sensyne Health and RH reported grants and personal fees from AstraZeneca, personal fees from GSK, personal fees from TEVA, personal fees from Amgen, personal fees from Astellas, personal fees from Abbvie, personal fees from Novartis, from Allergan, outside the submitted work; and is a director of Spoonful of Sugar Ltd, a University College London Business providing consultancy on treatment engagement and patient support programmes to healthcare policy makers, providers and industry.

For peer review only

Data

Dissemination

A manuscript with the results of the primary outcome will be published in a peer-reviewed journal. Additional manuscripts will report secondary outcomes and be submitted for publication in peer-reviewed journals.

Data availability

Data will be available from the main trial following completion of the analysis specified in the trial statistical analysis plan on request from the trial data repository (www.SuMMiT-D.org).

References

1. World Health Organization. Global report on diabetes. Geneva: World Health Organization; 2016.

2. UK D. Us, diabetes and a lot of facts and stats. London: Diabetes UK; 2019.

3. UK Prospective Diabetes Study Group. Overview of 6 years' therapy of type II diabetes: a progressive disease. Diabetes. 1995;44:1249–58.

4. Bommer C, Sagalova V, Heesemann E, Manne-Goehler J, Atun R, Bärnighausen T, et al. Global Economic Burden of Diabetes in Adults: Projections From 2015 to 2030. Diabetes Care. 2018 Apr 20;41(5):963–70.

5. UK Prospective Diabetes Study UKPDS Group. Efficacy of atenolol and captopril in reducing risk of macrovascular and microvascular complications in type 2 diabetes: UKPDS 39. 1998;317:713–20.

6. Colhoun H, Betteridge D, Durrington P, Hitman G, Neil H, Livingstone S, et al. Primary prevention of cardiovascular disease with atorvastatin in type 2 diabetes in the Collaborative Atorvastatin Diabetes Study (CARDS): multicentre randomised placebo-controlled trial. Lancet. 2004;364(1474-547; 9435):685–96.

7. Norris SL, Lau J, Smith SJ, Schmid CH, Engelgau MM. Self-Management Education for Adults With Type 2 Diabetes: A meta-analysis of the effect on glycemic control. 2002;25(7):1159–71.

8. McSharry J, McGowan L, Farmer AJ, French DP. Perceptions and experiences of taking oral medications for the treatment of Type 2 diabetes mellitus: a systematic review and meta-synthesis of qualitative studies. Diabet Med. 2016 Oct;33(10):1330–8.

9. Asche C, LaFleur J, Conner C. A Review of Diabetes Treatment Adherence and the Association with Clinical and Economic Outcomes. Clin Ther. 2011 Jan;33(1):74–109.

For peer review only

10. Salas M, Hughes D, Zuluaga A, Vardeva K, Lebmeier M. Costs of Medication Nonadherence in Patients with Diabetes Mellitus: A Systematic Review and Critical Analysis of the Literature. Value Health. Blackwell Publishing Inc; 2009 Sep 1;12(6):915–22.

11. Academy of Medical Sciences. “Patient adherence to medicines.” London; 2015. .

12. Zullig LL, Peterson ED, Bosworth HB. Ingredients of successful interventions to improve medication adherence. JAMA : the journal of the American Medical Association. American Medical Association; 2013 Dec 25;310(24):2611–2.

13. Nieuwlaat R, Wilczynski N, Navarro T, Hobson N, Jeffery R, Keepanasseril A, et al. Interventions for enhancing medication adherence. Nieuwlaat R, editor. Cochrane Database SystRev. Chichester, UK: John Wiley & Sons, Ltd; 2014;11:CD000011.

14. Ricci-Cabello I, Bobrow K, Islam SMS, Chow CK, Maddison R, Whittaker R, et al. Examining Development Processes for Text Messaging Interventions to Prevent Cardiovascular Disease: Systematic Literature Review. JMIR mHealth and uHealth. 2019 Mar 29;7(3):e12191.

15. Farmer AJ, McSharry J, Rowbotham S, McGowan L, Ricci-Cabello I, French DP. Effects of interventions promoting monitoring of medication use and brief messaging on medication adherence for people with Type 2 diabetes: a systematic review of randomized trials. Diabet Med. 2015 Oct 15;33(5):565–79.

16. Chow CK, Redfern J, Hillis GS, Thakkar J, Santo K, Hackett ML, et al. Effect of Lifestyle-Focused Text Messaging on Risk Factor Modification in Patients With Coronary Heart Disease: A Randomized Clinical Trial. JAMA : the journal of the American Medical Association. American Medical Association; 2015 Sep 22;314(12):1255–63.

17. Bobrow K, Farmer AJ, Springer D, Shanyinde M, Yu L-M, Brennan T, et al. Mobile Phone Text Messages to Support Treatment Adherence in Adults With High Blood Pressure (SMS-Text Adherence Support [StAR]): A Single-Blind, Randomized Trial. Circulation. 2016 Feb 9;133(6):592–600.

18. Kassavou A, Sutton S. Automated telecommunication interventions to promote adherence to cardio-metabolic medications: meta-analysis of effectiveness and meta-regression of behaviour change techniques. Health Psychology Review. Taylor & Francis; 2017 Sep 12;12(1):25–42.

19. Michie S, Richardson M, Johnston M, Abraham C, Francis J, Hardeman W, et al. The Behavior Change Technique Taxonomy (v1) of 93 Hierarchically Clustered Techniques: Building an International Consensus for the Reporting of Behavior Change Interventions. Ann Behav Med. 2013 Mar 20;46(1):81–95.

20. Long H, Bartlett YK, Farmer AJ, French DP. Identifying Brief Message Content for Interventions Delivered via Mobile Devices to Improve Medication Adherence in People With Type 2 Diabetes Mellitus: A Rapid Systematic Review. J Med Internet Res. 2019;21(1):e10421–19.

For peer review only

21. Horne R, Weinman J. Patients' beliefs about prescribed medicines and their role in adherence to treatment in chronic physical illness. J Psychosom Res. 1999;47(6):555–67.

22. Venkatesh V, Davis FD. A Theoretical Extension of the Technology Acceptance Model: Four Longitudinal Field Studies. Management Science. 2000 Mar 13;46(2):186–204.

23. Chan A-W, Tetzlaff JM, Altman DG, Laupacis A, Gøtzsche PC, Krleža-Jerić K, et al. SPIRIT 2013 statement: defining standard protocol items for clinical trials. American College of Physicians; 2013. pp. 200–7.

24. De Geest S, Zullig LL, Dunbar-Jacob J, Helmy R, Hughes DA, Wilson IB, et al. ESPACOMP Medication Adherence Reporting Guideline (EMERGE). Ann Intern Med. 2018 Jul 3;169(1):30–5.

25. Vrijens B, De Geest S, Hughes DA, Przemyslaw K, Demonceau J, Ruppar T, et al. A new taxonomy for describing and defining adherence to medications. Br J Clin Pharmac. 2012 Apr 5;73(5):691–705.

26. Bartlett K, Farmer A, Rea R, French DP. Developing acceptable brief messages based on behaviour change techniques (BCTs) to encourage medication adherence in people with Type 2 diabetes.

27. Herdman M, Gudex C, Lloyd A, Janssen M, Kind P, Parkin D, et al. Development and preliminary testing of the new five-level version of EQ-5D (EQ-5D-5L). Qual Life Res. Springer Netherlands; 2011 Dec;20(10):1727–36.

28. Schwarzer R. Modeling health behavior change: How to predict and modify the adoption and maintenance of health behaviors. Applied Psychology: an International Review. 57(1):1–29.

29. Curtis L, Burns A. Unit Costs of Health and Social Care 2018. Canterbury; 2018.

30. Joint Formulary Committee. British National Formulary. 77 ed. London.

31. Devlin NJ, Shah KK, Feng Y, Mulhern B, Ben van Hout. Valuing health-related quality of life: An EQ-5D-5L value set for England. Health Econ. John Wiley & Sons, Ltd; 2018 Jan 1;27(1):7–22.

32. Braun V, Clarke V. Using thematic analysis in psychology. Qualitative Research in Psychology. 2006 Jan;3(2):77–101.

33. May C. Towards a general theory of implementation. Implementation Science. Implementation Science; 2013 Feb 13;8(1):1–1.

34. Tabachnik BG, Fidell LS. Using multivariate statistics. 6 ed. London: Pearson; 1996.

35. Nguyen T-M-U, Caze AL, Cottrell N. What are validated self-report adherence scales really measuring?: a systematic review. Br J Clin Pharmac. 2014 Feb 21;77(3):427–45.

For peer review only - http://bmjopen.bmj.com/site/about/guidelines.xhtml

36. Harris PA, Taylor R, Thielke R, Payne J, Gonzalez N, Conde JG. Research electronic data capture (REDCap)—A metadata-driven methodology and workflow process for providing translational research informatics support. Journal of Biomedical Informatics. Elsevier Inc; 2009 Apr 1;42(2):377–81.

For peer review only

Figure 1

Trial Profile

Figure 2

Logic model

Title: Logic model showing anticipated mechanisms of action in the SuMMIT-D Feasibility study, based on the Health Action Process Approach

(a) *Footnote*: (a) This construct includes both beliefs about the necessity of taking medication, and concerns about taking that medication.

For peer review only

Table 1

Schedule of trial outcomes and measures

Outcome	Measure	Timing (months)		
		0	6	18
Participant recruitment to the trial.	Recruitment against planned recruitment rates.	x		
Participant willingness to be randomised.	Number of people showing an interest and not proceeding or those who withdraw from the control group and give a reason	x		
Feasibility of collection of clinical measurement data for the proposed clinical trial.	Retrieval of measurements of HbA1c, systolic blood pressure and cholesterol for trial participants from medical record.	x	x	
Willingness of participants to be followed up over the 26-week period post randomisation.	Retention and follow-up rates		x	
Feasibility of collection of prescribing data on trial participants.	Proportion of medication possession ratio for glucose, blood pressure and lipid lowering medication obtainable from prescribing data in participant's medical record		x	
Feasibility of collecting self-reported questionnaire measures.	Proportion of completed self-reported measures (Demographics and additional information Questionnaire; MARS self-report scale; EQ-5D-5L Health status questionnaire; Healthcare Utilisation Record Questionnaire (cost measurement); Health psychology and technology acceptance questionnaire.	x	x	
Feasibility of collecting medical history and baseline medication from medical record	Proportion of data obtained	x		
Feasibility and acceptability of the intervention for patients and healthcare professionals (including general practitioners, nurses, receptionists and pharmacists). Qualitative process evaluation	Data obtained through focus groups, qualitative interviews with patients and healthcare staff.	x	x	
Assess reliability of measures of hypothesised mechanism of action, and sensitivity to change in response to receipt of SMS messages. Examine relationship between these measures and self-reported adherence, as preliminary process analysis.. (Quantitative process evaluation)	Change in quantitative mechanism of action measures and relationship between changes in these measures and self-reported adherence.	x	x	
Changes in clinical measurement data	HbA1c, systolic blood pressure and cholesterol for trial participants from medical record.	x	x	x
Information on message delivery and interaction with participants.	Automated reports from messaging service on messages delivered and interactive messaging	x	x	

For peer review only

For peer review only

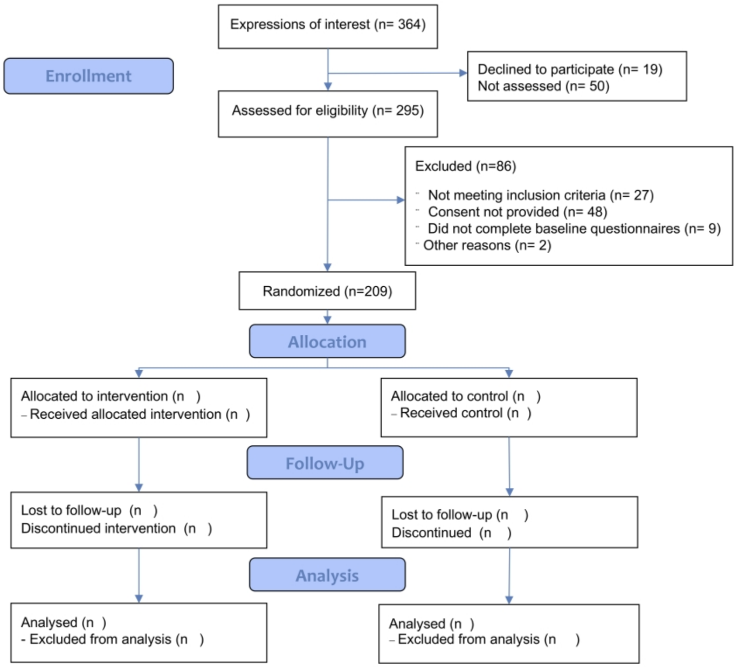

Figure 1 Trial Profile

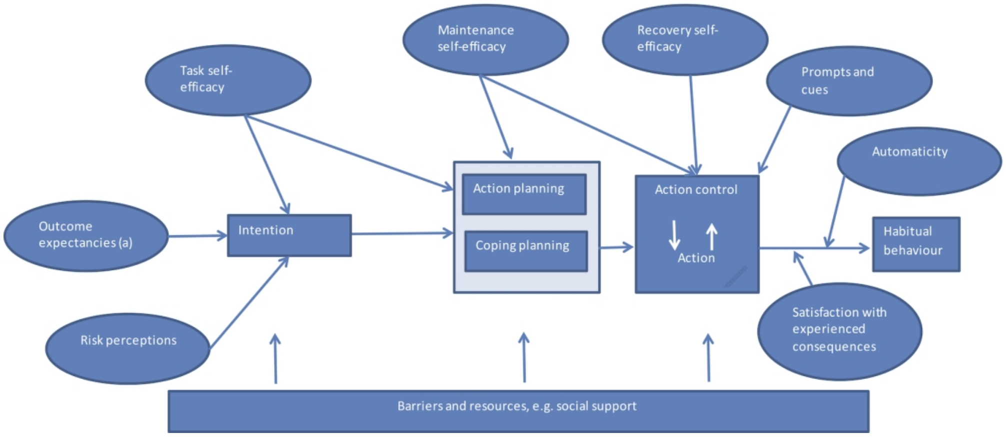

Figure 2: Logic Model

Supplementary Appendix

This appendix has been provided by the authors to give readers additional information about their work.

Supplement to: Supporting people with type 2 diabetes in effective use of their medicine through a system comprising mobile health technology integrated with clinical care compared with usual care (SuMMiT-D Feasibility): a randomised feasibility trial protocol

For peer review only

Appendix 1.

Examples of SMS text messages used in SuMMiT-D feasibility

Target and category of message	BCT/ Belief or concern	Example messages
Medication adherence, BCT	1.4 Action Planning	Plan when, where and how you are going to take your medication.
Medication adherence, BCT	15.1 Verbal persuasion about capability	If you are struggling with your diabetes tablets then don't worry, you will be able to master it in time. You will get on top of it.
Medication adherence, BCT	7.1 Prompts/ cues	It can be difficult to remember to take your tablets. Why not set an alarm to remind you to take them?
Medication adherence, BCT	2.3 Self-monitoring	Find a way to split your tablets into days so you notice when you have forgotten to take your tablets
Medication adherence, BCT	3.2 Social support (practical)	How often do you forget to take your tablets? Take control. Ask your friends and family members to help. Their reminders could help you to improve your diabetes
Medication adherence, BCT	15.2 Mental rehearsal of successful performance	Visualise in detail how you will take your tablets tomorrow. This will make it easier when you actually take them
Medication adherence, BCT	3.3 Social support (emotional)	If you're not taking your tablets as often as you should, try discussing your feelings with someone.
Medication adherence, BCT	15.2 Mental rehearsal of successful performance	Think about situations where taking tablets was easy. How could you make your everyday tablet taking like this?
Medication adherence, beliefs and concerns	G Healthcare system related concerns	Lots of questions? Check who the best person to see might be
Diet management	Signposting	Stuck for new ideas? You can search recipes for mains, desserts and snacks online at Diabetes.org.uk

Glossary: BCT- Behaviour Change Technique

For peer review only - http://bmjopen.bmj.com/site/about/guidelines.xhtml

Appendix 2 Beliefs and Ideas questionnaire

For peer review only

Participant ID: ___ ___ / ___ ___ ___ / ___ ___ ___ ___

Date Completed: DD / MMM / YYYY

SuMMiT-D Feasibility

Ideas and concerns about your diabetes treatment – Baseline

(Questionnaire to be adapted for electronic use as well)

This survey is about what you think and feel about your diabetes tablets, as well as how you approach taking them. There are no right or wrong answers. We know some of the items might be difficult to answer, but please choose the answer that best describes what you think. When you are completing the survey please think about the tablets you take for your diabetes.

		Strongly disagree	Disagree	Neither agree nor disagree	Agree	Strongly Agree
1.	It is easy to take my diabetes tablets as prescribed	☐	☐	☐	☐	☐
2.	I am confident that I can take my diabetes tablets as prescribed	☐	☐	☐	☐	☐
3.	Without my diabetes tablets I would be very ill	☐	☐	☐	☐	☐
4.	My health in the future will depend on my diabetes tablets	☐	☐	☐	☐	☐
5.	I sometimes worry about the long-term effects of my diabetes tablets	☐	☐	☐	☐	☐
6.	My diabetes tablets are a mystery to me	☐	☐	☐	☐	☐
7.	I want to take my diabetes tablets as prescribed every day over the next 3 months	☐	☐	☐	☐	☐
8.	I will take my diabetes tablets as prescribed every day over the next 3 months	☐	☐	☐	☐	☐

For peer review only - http://bmjopen.bmj.com/site/about/guidelines.xhtml

Participant ID: ___ ___ / ___ ___ ___ / ___ ___ ___ ___

		Strongly disagree	Disagree	Neither agree nor disagree	Agree	Strongly Agree
9.	Taking my diabetes tablets as prescribed is something I do without thinking.	☐	☐	☐	☐	☐
10.	Taking my diabetes tablets as prescribed is something I do without noticing that I do	☐	☐	☐	☐	☐
11.	I am confident that I am able to take my diabetes tablets as prescribed even when something disrupts my routine	☐	☐	☐	☐	☐
12.	I am confident that I can take my diabetes tablets as prescribed even when I feel well	☐	☐	☐	☐	☐
13.	If I don't take my diabetes tablets for any reason, I am confident that I am able to start taking them again even if I feel no different to when I was not taking them	☐	☐	☐	☐	☐
14.	If I don't take my diabetes tablets for any reason, I can start taking them again even if I had not taken them for a couple of days	☐	☐	☐	☐	☐
15.	I have made a detailed plan for when to take my diabetes tablets	☐	☐	☐	☐	☐
16.	I have made a detailed plan about exactly where to take my diabetes tablets	☐	☐	☐	☐	☐
17.	I have made a detailed plan for what to do to help me take my diabetes tablets as prescribed when facing barriers to do so	☐	☐	☐	☐	☐

For peer review only - http://bmjopen.bmj.com/site/about/guidelines.xhtml

Participant ID: ___ ___ / ___ ___ ___ / ___ ___ ___ ___

		Strongly disagree	Disagree	Neither agree nor disagree	Agree	Strongly Agree
18.	I have made a detailed plan for how to deal with unpleasant side effects of taking my diabetes tablets as prescribed	☐	☐	☐	☐	☐
19.	During the last 4 weeks I consistently monitored when, where, and how I took my diabetes tablets	☐	☐	☐	☐	☐
20.	During the last 4 weeks I really tried hard to take my diabetes tablets as prescribed	☐	☐	☐	☐	☐
21.	I use things around me to help me to take my diabetes tablets as prescribed (e.g. notes, phone reminders)	☐	☐	☐	☐	☐
22.	I change things around me to prompt me to take my diabetes tablets as prescribed	☐	☐	☐	☐	☐
23.	I have had somebody help me to take my diabetes tablets as prescribed	☐	☐	☐	☐	☐
24.	I have felt supported in taking my diabetes tablets as prescribed	☐	☐	☐	☐	☐
25.	Given the effort I have put into taking my diabetes tablets, I am content with my diabetes control	☐	☐	☐	☐	☐
26.	I am content with what I have experienced as a result of taking my diabetes tablets	☐	☐	☐	☐	☐
27.	It is likely that I will develop complications, or experience	☐	☐	☐	☐	☐

For peer review only - http://bmjopen.bmj.com/site/about/guidelines.xhtml

Participant ID: ___ ___ / ___ ___ ___ / ___ ___ ___ ___

		Strongly disagree	Disagree	Neither agree nor disagree	Agree	Strongly Agree
	worse complications from my diabetes if do not take my tablets					
28.	I feel very at risk of developing complications, or experiencing worsening of existing complications from my diabetes if I do not take my tablets	☐	☐	☐	☐	☐

If you have any comments about this survey, please leave them below.

Thank you for completing this survey.

Please return to the SuMMiT-D Feasibility study team in the envelope provided.

If you have any questions, please contact: xxxxxxxxxxx

For peer review only - http://bmjopen.bmj.com/site/about/guidelines.xhtml

Appendix 3

Members of the SuMMiT-D Feasibility Trial Collaborative Group:

Writing Group

A. J. Farmer[1], J.Allen[1], Y.K. Bartlett[2], P. Bower[2], Y. Chi[1], D.P. French[2], B Gudgin[3], E. Holmes[4], R. Horne[5], D.A. Hughes[4], C. Kenning[2], L. Locock[6], J. McSharry[7], L. Miles[2], N. Newhouse[1], R. Rea[1], E. Riga[1], L. Tarassenko[1], C. Velardo[1], N. Williams[1], V. Williams[1], L-M Yu[1].

Author affiliations:
1. University of Oxford, Oxford UK.
2. University of Manchester, Manchester UK.
3. Patient Representative, Oxfordshire, UK.
4. Bangor University, Bangor UK.
5. University College London, UK.
6. University of Aberdeen, Aberdeen UK
7. National University of Ireland, Galway, Eire.

Programme Steering Committee

Independent Members

Chair: K. Khunti; C. Weir, F. Sniehotta, S. England

Investigators

A. Farmer; D.P. French, L-M. Yu

Observers

T. Hutchinson (Funder), E. Chick (Sponsor), C. Velardo, E. Riga, S. M. Robinson

Trial Management Team

J. Allen, Senior Trial Manager; E. Riga, Trial Manager; S. M. Robinson, Trial Coordinator; C. Kenning, Regional Coordinator

Statistics

L-M. Yu, Lead Trial Statistician; N. Williams, Senior Statistician; M. Shanyinde, Statistician

Data Management

S. Kelly, Senior Data Manager; L. Engonidou, Data Manager

IT, Programming and Engineering

D. Judge, Senior IT and Programming Manager; C. Velardo, Senior Research Fellow; Y. Chi, Post-doctoral Research Assistant.

Research Nurses

H. Rutter, Senior Research Nurse; S. Squires, Research Nurse; B. Mundy, Research Nurse; K. Madronal, Research Nurse; I. Noel, Research Nurse; B. I'Anson, Research Nurse; P. Whitbread, Research Nurse

PPI Members

Chair: B. Gudgin; G. Moultrie, M. Simpson, P. Baller, R. Dawett, D. Akker, M. Mistry, S. Mulholland, J. Nugent, G. Sides, D. Parkinson, S. McPhillips

Acknowledgements
In addition to the team members acknowledged above, we would also like to acknowledge the help of the following clinical research networks (CRNs) and participating GP practices supporting recruitment:

Practice Name
NIHR CRN: Thames Valley & South Midlands
South Oxford Health Centre
White Horse Medical Practice
Botley Medical Centre
The Rycote Practice
Milman Road Health Centre/Milman Medical
Broadshires Health Centre
Bicester Health Centre
NIHR CRN: West Midlands
Eve Hill Medical Practice
Spring Gardens Group Medical Practice
Burbury Medical Centre
NIHR CRN: South West Peninsula
Brunel Medical Practice
Newquay Health Centre
Glastonbury Surgery
NIHR CRN: Greater Manchester
Block Lane Surgery
Ashcroft Surgery
West Timperley Medical Centre
Chorlton Family Practice
Washway Road Medical Centre
Market Street Medical Practice

Trial Sponsor: University of Oxford. Contact Clinical Trials and Research Governance, Churchill Hospital, Oxford, UK.

For peer review only

TIDieR checklist for the Supporting people with type 2 diabetes in effective use of their medicine through mobile health technology integrated with clinical care (SuMMiT-D) feasibility trial

**1.**	**Brief name**	Supporting people with type 2 diabetes in effective use of their medicine through mobile health technology integrated with clinical care (SuMMiT-D Feasibility)	
**2.**	**Rationale or theory**	Systematic reviews of text messages to support patients to adhere to treatment, and of mobile health interventions in diabetes, identify some effective interventions. There are a few trials testing the impact of brief messaging in type 2 diabetes, but they do not have systematically developed interventions based on theory and evidence and are at risk of bias.[1,2] Recent trials of text-messaging for cardiovascular risk prevention and blood pressure lowering have shown clinically relevant changes in outcomes compared with usual care.[3,4]<br>In addition, there is substantial evidence[5] that tailored interventions are more effective than generic interventions. Tailored interventions may be seen by recipients as more personally relevant, so they will be more likely to attend to, read, understand, and act on them. In addition, tailored interventions are designed to change determinants of the target behaviour that are relevant to particular individuals or to small subgroups of individuals; they therefore more precisely target the determinants of the individual's behaviour.<br>SuMMiT-D (SUpport through Mobile Messaging and digital health Technology for Diabetes) is a programme of work comprised of three phases: formative work, a feasibility trial and a large scale, effectiveness randomised controlled trial of a mobile phone-based system intended to deliver brief, tailored, behaviour-change messages to people with type 2 diabetes focusing on use of medication, lifestyle and other aspects of diabetes management. In the formative work for this trial, we identified theoretical constructs and features of intervention content found to be associated with medication adherence in patients with type 2 diabetes and mapped these onto a standard taxonomy for behaviour change techniques (BCTs), that is, active ingredients of interventions used to promote behavioural change[6,7]. We then developed a large set of messages to target each BCT, and examined which types of messages are most useful and easy to understand for people starting and taking an oral diabetes medicine, and the extent to which it might be helpful for patients to decide on the types of messages they want to receive. We received input from approximately 300 patients with type 2 diabetes and healthcare professionals caring for this patient population. Preliminary findings of this trial development work were used to develop this feasibility trial with scope for further refinement of the system for the main clinical trial.	
		Usual care and one non-health-related message a month	Condition-specific tailored text messaging system and usual care
		Active control: Infrequent non-health related messages sent to all participants,<br><br>The purpose of the infrequent messages is to:<br>i) Maintain participant interest in the trial.<br>ii) Make it less clear who was getting which intervention	i. Participants will be sent up to four automated text-messages per week with an average frequency of three per week relating to diabetes management and use of medicine.<br>ii. The library of text-messages uses different behaviour change techniques to target health-related behaviour change relating to use of medicines, as well as messages targeting other aspects of diabetes care (including diet and exercise)..

For peer review only - http://bmjopen.bmj.com/site/about/guidelines.xhtml

		iii) Exclude receipt of "any SMS" as effecting health-related behaviour	iii. Frequency of messages received using a particular type of behaviour change technique can be modified based on a participant's response to individual messages received.<br>The style of messages will be patient-centred and will encourage patients to seek further relevant information (including the use of links where possible to selected external websites e.g. Diabetes UK).
**3.**	**Materials**	Available health materials on type 2 diabetes routinely provided by the health care service. All participants were sent a system user guide by email.	
**4.**	**Procedures**	1. Language and timing of messages selected by participant<br>2. Types of brief messages<br>a) Welcome message (confirming sign-up)<br>b) Message with options for changing delivery time<br>c) Non-health related messages sent every 4 weeks for 6-months<br>d) Thank you message at end of study	1. Timing of messages selected by participant<br>2. Types of brief messages<br>a) Welcome messages (confirming sign-up)<br>b) Message with instructions about use of system<br>c) Brief treatment and lifestyle adherence support messages randomly selected from library (with rules that ensured individual messages were not repeated) and sent several times per week, each week for 6-months<br>d) Reminders of how to use the system to "Like" and "Dislike" messages, STOP and PAUSE sending of messages<br>e) Thank you message at end of study
**5.**	**Intervention provider**	Automated SMS text-message delivery platform using open-source software and third party bulk SMS-delivery provider	
**6.**	**Modes of delivery**	Intervention delivered via 160 characters SMS-text sent to individual participant's own handset. Initial message sent to all enrolled participants is Welcome message confirming sign-up, thereafter after automated messages sent to individual participants as per randomised allocation for 6-months.	
**7.**	**Location where intervention occurred**	Community	
**8.**	**Number of times intervention was delivered over what time period**	Brief trial related and non-health messages sent once every 4 weeks for 6-months	Brief messages sent three (min) to four (max) times per week for 6-months
**9.**	**What, why, when, how intervention was personalised or adapted**	1. Timing of messages selected by participant.	1. Timing of messages selected by participant<br>2. The group (behavioural change technique) from which messages are sent is modified on the basis of the

For peer review only - http://bmjopen.bmj.com/site/about/guidelines.xhtml

			participant sending "likes" and "dislikes" as text-messages
**10.**	**Modifications during the trial**	Nil	Nil
**11.**	**Planned intervention delivery**	Brief messages will be sent using an automated system independent of trial and clinical staff. Participants will be told that not everyone will be receiving the exact same messages. Participants will also be asked not to share their health messages with others. Intervention fidelity (receipt of messages and linkage to an individual) will be checked by response to the REGISTER request. Message delivery reports will be monitored throughout the trial to check the intervention is being delivered as planned. Messages not delivered (network unavailable etc.) will be resent up to three times. Where message delivery fails after three sequential attempts a failed message protocol will be initiated to track the participant and up-date their phone number if required.	
**12.**	**Actual intervention delivery**	Will be monitored during trial, blind to allocated group	

1. Ricci-Cabello I, Bobrow K, Islam SMS, Chow CK, Maddison R, Whittaker R, et al. Examining Development Processes for Text Messaging Interventions to Prevent Cardiovascular Disease: Systematic Literature Review. JMIR mHealth and uHealth. 2019 Mar 29;7(3):e12191.
2. Farmer AJ, McSharry J, Rowbotham S, McGowan L, Ricci-Cabello I, French DP. Effects of interventions promoting monitoring of medication use and brief messaging on medication adherence for people with Type 2 diabetes: a systematic review of randomized trials. Diabet Med. 2015 Oct 15;33(5):565–79.
3. Chow CK, Redfern J, Hillis GS, Thakkar J, Santo K, Hackett ML, et al. Effect of Lifestyle-Focused Text Messaging on Risk Factor Modification in Patients With Coronary Heart Disease: A Randomized Clinical Trial. JAMA : the journal of the American Medical Association. American Medical Association; 2015 Sep 22;314(12):1255–63.
4. Bobrow K, Farmer AJ, Springer D, Shanyinde M, Yu L-M, Brennan T, et al. Mobile Phone Text Messages to Support Treatment Adherence in Adults With High Blood Pressure (SMS-Text Adherence Support [StAR]): A Single-Blind, Randomized Trial. Circulation. 2016 Feb 9;133(6):592–600.
5. Kassavou A, Sutton S. Automated telecommunication interventions to promote adherence to cardio-metabolic medications: meta-analysis of effectiveness and meta-regression of behaviour change techniques. Health Psychology Review. Taylor & Francis; 2017 Sep 12;12(1):25–42.
6. Michie S, Richardson M, Johnston M, Abraham C, Francis J, Hardeman W, et al. The Behavior Change Technique Taxonomy (v1) of 93 Hierarchically Clustered Techniques: Building an International Consensus for the Reporting of Behavior Change Interventions. Ann Behav Med. 2013 Mar 20;46(1):81–95.
7. Long H, Bartlett YK, Farmer AJ, French DP. Identifying Brief Message Content for Interventions Delivered via Mobile Devices to Improve Medication Adherence in People With Type 2 Diabetes Mellitus: A Rapid Systematic Review. J Med Internet Res. 2019;21(1):e10421–19.

For peer review only - http://bmjopen.bmj.com/site/about/guidelines.xhtml
